# Plastid phylogenomics with broad taxon sampling further elucidates the distinct evolutionary origins and timing of secondary green plastids

**DOI:** 10.1038/s41598-017-18805-w

**Published:** 2018-01-24

**Authors:** Christopher Jackson, Andrew H. Knoll, Cheong Xin Chan, Heroen Verbruggen

**Affiliations:** 1 School of Biosciences, University of Melbourne, Melbourne, Victoria 3010 Australia; 2 Department of Organismic and Evolutionary Biology, Harvard University, Cambridge, Massachusetts 02138 USA; 3 Institute for Molecular Bioscience, and School of Chemistry and Molecular Biosciences, The University of Queensland, Brisbane, Queensland 4072 Australia

## Abstract

Secondary plastids derived from green algae occur in chlorarachniophytes, photosynthetic euglenophytes, and the dinoflagellate genus *Lepidodinium*. Recent advances in understanding the origin of these plastids have been made, but analyses suffer from relatively sparse taxon sampling within the green algal groups to which they are related. In this study we aim to derive new insights into the identity of the plastid donors, and when in geological time the independent endosymbiosis events occurred. We use newly sequenced green algal chloroplast genomes from carefully chosen lineages potentially related to chlorarachniophyte and *Lepidodinium* plastids, combined with recently published chloroplast genomes, to present taxon-rich phylogenetic analyses to further pinpoint plastid origins. We integrate phylogenies with fossil information and relaxed molecular clock analyses. Our results indicate that the chlorarachniophyte plastid may originate from a precusor of siphonous green algae or a closely related lineage, whereas the *Lepidodinium* plastid originated from a pedinophyte. The euglenophyte plastid putatively originated from a lineage of prasinophytes within the order Pyramimonadales. Our molecular clock analyses narrow in on the likely timing of the secondary endosymbiosis events, suggesting that the event leading to *Lepidodinium* likely occurred more recently than those leading to the chlorarachniophyte and photosynthetic euglenophyte lineages.

## Introduction

The spread of plastids by secondary endosymbiosis – the uptake of an alga containing a primary plastid by a heterotrophic eukaryotic host – has driven the evolution of many photosynthetic lineages of global ecological and economic importance. Haptophytes, diatoms and most photosynthetic dinoflagellates, for example, contain red-algal derived plastids originating from secondary (or subsequent higher) endosymbiotic events^1–3^, and together these organisms constitute major primary producers in marine environments. Other lineages contain a secondary plastid originating from a green algal ancestor^4–7^. Due in part to their ubiquity and diversity^8,9^, lineages with secondary red plastids have undergone intense scrutiny, whereas organisms with secondary green plastids are less well-studied.

Currently, three lineages are known to contain secondary plastids derived from green algae: euglenophytes, chlorarachniophytes and the dinoflagellate genus *Lepidodinium*^10^. Photosynthetic euglenophytes occur in both freshwater and marine environments^11^, and these taxa and their secondarily heterotrophic descendants comprise the class Euglenophyceae, forming a clade within the broader Euglenida *sensu* Adl *et al*.^12^. Chlorarachniophytes, a small group of exclusively marine unicellular algae, are often amoeboid in form and belong to the supergroup Rhizaria. Together with cryptophytes they are unique among lineages with secondary plastids in that they retain a vestigial nucleus from the algal endosymbiont, termed a nucleomorph^13,14^. Dinoflagellates are a large group of flagellated marine and freshwater protists belonging to the supergroup Alveolata. Approximately half are photosynthetic, harbouring a secondary plastid derived from a red algal ancestor, and these plastids contain the pigment peridinin^15^. Some dinoflagellate lineages, however, have subsequently acquired a plastid from a different source through additional secondary or tertiary endosymbioses (the latter involving uptake of an alga already containing a secondary plastid)^16^. Yet other species contain temporary but functional kleptoplastids (stolen plastids) acquired from algal prey^17,18^. The genus *Lepidodinium* contains a secondary plastid derived from a green algal ancestor. Two species are currently recognised, *L. viride* and *L. chlorophorum*^19^. Given that the secondary peridinin-type plastid is present in the last common dinoflagellate ancestor, the green *Lepidodinium* plastid is postulated to have originated from an additional secondary event via so-called ‘serial secondary endosymbiosis’^16,20^.

While there is ongoing controversy about exactly how many secondary (and perhaps higher) endosymbiosis events have led to the numerous lineages containing a secondary red plastid^21^, the situation for secondary green plastids is more clear-cut, and it is now apparent that euglenophytes, chlorarachniophytes and *Lepidodinium* acquired their plastids in three independent evolutionary events. Moreover, phylogenies of plastid genes recover the three lineages branching with different, relatively unrelated groups of green algae, indicating a distinct plastid origin in each case^4,5,22^. For *Lepidodinium*, recent phylogenomic analyses of the plastid genome (plDNA) recovered a well-supported sister relationship with the pedinophyte *Pedinomonas minor*^4^, whereas euglenophyte plastids are sister to prasinophytes from the order Pyramimonadales^22^. The ancestor of the chlorarachniophyte plastid remains uncertain^23^; although recent plDNA phylogenies recovered a well-supported relationship between chlorarachniophyte plastids and the green-algal order Bryopsidales, the closest relative of this clade within the green algal phylogeny was not resolved^5^. Overall, analyses of the origin of secondary green plastids suffer from relatively sparse taxon sampling within the green algal groups to which they are related. In recent years, complete plastid genomes (plDNA) from diverse green algae have been sequenced, encouraging us to revisit the question.

In this study we use an expanded dataset of publically available sequences, together with strategically selected newly sequenced plastid genomes, to re-examine the origins of secondary green plastids using a phylogenomics approach. In doing so, we aim to pinpoint the closest extant relatives of the ancestral green algae that were involved in the three distinct endosymbiosis events, and to infer the timing of these events using relaxed molecular clock methods.

## Materials and Methods

### Culturing, DNA extraction, sequencing, plDNA assembly, and gene annotation

The pedinophyte strain YPF-701 (NIES Microbial Culture Collection strain NIES-2566) was cultured in Guillard’s F/2 media at 20 °C on a 14-h light/10-h dark cycle. *Avrainvillea mazei* HV02664 (representing the early-branching family *Dichotomosiphonaceae* of the Bryopsidales) and *Neomeris* sp. HV02668 (representing the *Dasycladaceae*, an early-branching Dasycladales family) were collected from Tavernier (FL, USA) and Islamorada (FL, USA), respectively, and preserved in silica-gel. For collection details of *Ostreobium* sp. HV05042 see^24^. For pedinophyte YPF-701 cells were harvested by centrifugation (10 min, 3,000 *g*). Total genomic DNA was extracted using a modified CTAB protocol^25^, in which the CTAB extraction buffer was added directly to the cell pellet. Due to technology availability and variation of DNA yields, two sequencing strategies were used. For *Avrainvillea mazei* HV02664, a TruSeq Nano LT library (~350 bp inserts) was prepared for sequencing of 2 × 100 bp paired-end reads using the Illumina HiSeq 2000 platform. For the other two strains, libraries (~500 bp inserts) were prepared using a Kapa Biosystems kit for sequencing of 2 × 150 bp paired-end reads using the Illumina NextSeq platform. All libraries were sent for sequencing at Novogene (Hong Kong).

Sequence reads were assembled using SPAdes 3.8.1^26^ using the –careful option. Contigs matching to pedinophyte or Ulvophycean chloroplast genome reference sequences were imported into Geneious 9.1.3 (http://www.geneious.com), where completeness and circularity were manually evaluated. Final contigs were annotated following Verbruggen and Costa^27^ and Marcelino *et al*.^28^. In brief, annotations were obtained from MFannot, DOGMA and ARAGORN and imported into Geneious. All annotations were vetted manually and a master annotation track was created from them.

### Phylogenetic analyses

#### Maximum Likelihood phylogenies

All 151 chloroplast genomes for green algae, photosynthetic euglenophytes, *Lepidodinium chlorophorum* and chlorarachniophytes used in this study are shown in Table S7. For each protein-coding gene, protein sequences were aligned using MAFFT 7.215^29^, after which the aligned amino acid residues were reverse translated into the corresponding coding nucleotide sequences (in fixed codon positions) using TranslatorX^30^. Genes that were present in >50% of total taxa (64 genes) were included in subsequent analyses. For each alignment, poorly aligned regions were removed via an automated algorithm using the Gblocks software^31^ version 0.91b with options −t = c − b5 = h. Single-gene alignments were concatenated to produce a multigene supermatrix (Dataset A, 34,452 nucleotides) using Geneious (Biomatters) (see Supplementary Table S7 for missing data percentages), and an amino-acid translation of the nucleotide alignment was generated. The nucleotide alignment was partitioned by gene and codon position and Partition Finder^32^ was used to determine the best-fit partitioning scheme. Partition Finder was run multiple times, once for each of the following independent models: GTR, HKY, JC69, and K80. The amino-acid alignment was partitioned by gene, and Partition Finder was used to assign one of the following models to each partition: LG, WAG, MTREV, JTT, CPREV, DAYHOFF, BLOSUM62. For nucleotide analyses, individual maximum likelihood (ML) trees were estimated for each model/partitioning scheme, using the concatenated dataset with RAxML v8.2.6^33^ and 500 non-parametric bootstrap replicates. RAxML amino-acid analyses were also performed with 500 non-parametric bootstrap replicates. For both nucleotide and amino-acid analyses a gamma model of rate heterogeneity with four categories was used. For amino-acid analyses, empirical amino-acid frequencies were applied to partitions where recommended by Partition Finder.

For site-stripping analyses, per-site substitution rates were calculated for our Dataset A alignments using HyPhy^34^, and the fastest evolving sites were removed using SiteStripper v.1.01 (http://www.phycoweb.net/software/SiteStripper/index.html, last accessed November 24, 2016), leaving a percentage length of the original alignment (95%, 90%, 85%, 80%, 75%, 70%, 65%, 60%, 55%, 50%). ML trees were estimated with RAxML as above under a GTR model, retaining partitions, with 100 non-parametric bootstrap replicates.

In addition to analyses with Dataset A, we also performed ML analyses on a second dataset including some very recently released green-algal plDNAs as well as sequences recently generated in our laboratory (Dataset B, see Supplementary Table S9 for taxa list), to evaluate whether the new sequences affected the branching positions of secondary plastid lineages and hence our overall conclusions. The Dataset B nucleotide supermatrix (69 genes, 35,739 nucleotides) was generated as per above, and an amino-acid translation was produced. Trees were generated using RAxML (LG model for amino-acid analysis; GTR model with partitioning by codon position for nucleotide analysis), with 100 rapid bootstrap replicates. A tree was also estimated from the amino-acid alignment using IQ-TREE^35^ with the site-heterogeneous LG + G4 + C60 + F + PMSF model^36^, using the topology estimated by the RAxML amino-acid analysis as a guide tree to compute PMSF profiles.

#### Bayesian phylogenies

Bayesian analyses of both nucleotide and amino-acid alignments (Dataset A) were performed using Phylobayes 4.1b^37^ with the CATGTR model. Two independent Markov chain Monte Carlo runs were performed for each analysis for a total of 10,000 cycles. A consensus topology was calculated using the bpcomp program; the first 2,000 cycles from each chain were discarded as burn-in, trees from both chains were sampled every ten cycles, and a majority-rule posterior consensus tree was generated. For both nucleotide and amino-acid analyses the largest discrepancy observed across all bipartitions (maxdiff) was 1, indicating that at least one of the runs was stuck in a local maximum in each case.

### Relaxed molecular clock analyses

To select genes for the molecular clock analyses we followed the methods of Castresana (2000) using the SortaDate package, using the ML tree topology recovered in the amino-acid analyses (Dataset A) and amino-acid translations of single-gene alignments. A dataset was compiled from the top-ranked 11 genes (*tufA*, *rps7*, *rps4*, *rpl14*, *rpl5*, *rpl2*, *psbC*, *psbB*, *petA*, *atpI*, *atpA*). In addition, we included *atpB* and *rbcL* to allow testing of fossil calibrations from the Chlorophyceaen family Hydrodictyaceae. See Supplementary Information File S1 for full methods and details of node calibrations and chronogram analyses.

### Data availability

Plastid sequence data is deposited in Genbank under accession KY347917 (pedinophyte strain YPF-701), KY509314 (*Ostreobium* sp. HV05042), KY495826-KY495874 (*Neomeris* sp.), and KY509313 (*Avrainvillea mazei*). All other data generated or analysed during this study are either publically available or included in this published article (and its Supplementary Information files).

## Results and Discussion

**Figure 1:**
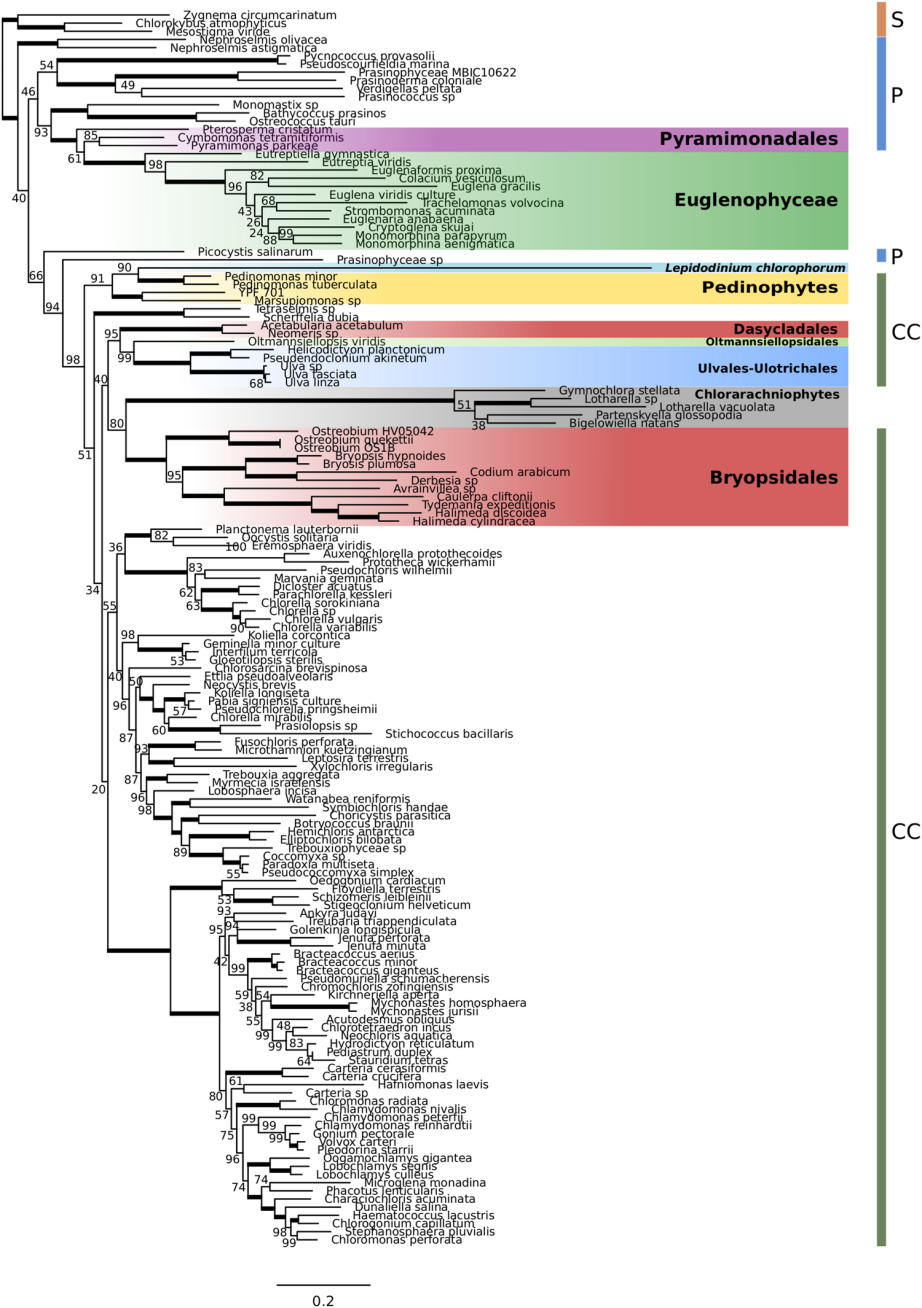
RAxML phylogenetic analysis inferred from an amino acid alignment of 64 plastid genes from streptophytes, green algae, photosynthetic euglenophytes, the “green” dinoflagellate *Lepidodinium chlorophorum*, and chlorarachniophytes. The dataset was partitioned by gene according to the best-fit model assigned by PartitionFinder. Thick branches have full ML bootstrap support. Coloured vertical bars to the right of the phylogeny are labelled: S, Streptophytes; P, prasinophytes; CC, core Chlorophyta. Branch lengths are proportional to the number of substitution per site.

To increase the density of taxon sampling in lineages potentially related to secondary endosymbionts, we sequenced plDNAs from lineages in the Ulvophyceae (*Ostreobium* sp. HV05042, complete; *Avrainvillea mazei*, near complete; *Neomeris* sp., fragmented) and Pedinophyceae (strain YPF-701, complete), and combined them with publically available complete or near-complete plastid genome data (see Supporting Information Table S7). Accelerated rates of evolution have been observed in secondary green plDNAs, reflected in long branches in phylogenetic analyses^4,38^. This could potentially result in long-branch attraction (LBA), a phylogenetic artefact in which long branches cluster together even if they are not closely related^39^. Increased sampling of putatively related lineages minimises long branches in the clades which contain them, reducing potential LBA between secondary plastids and their closest green-algal relatives. Using our dataset of 151 taxa and 64 genes (Dataset A; see methods), we carried out Maximum likelihood (ML) and Bayesian analyses using both nucleotide and amino-acid alignments. Our phylogenies support the three independent gains of secondary green plastids (Fig. 1; see below for discussion of additional analyses, Supplementary Figs S1–S7). The topology of the backbone green-algal tree largely corresponds to previously published studies, with a series of early-branching prasinophyte lineages, a monophyletic “core Chlorophyta” and little support for the monophyly of traditionally recognized Ulvophyceae and Trebouxiophyceae^40^.

### Euglenophytes

Previous plastid phylogenomic studies have recovered a sister relationship between the Euglenophyceae and the prasinophyte order Pyramimonadales^7,22,41,42^. These studies, together with lines of evidence such as plDNA gene-linkage conservation^22^ and individual gene similarity^43^ between Euglenophyceae and pyramimonadalean taxa, suggest that the secondary endosymbiosis event leading to the Euglenophyceae involved a pyramimonadalean alga.

In this study, we have collated publically available data to include the most comprehensive taxon sampling of chloroplast genomes in both the Euglenophyceae (12 taxa) and Pyramimonadales (three taxa) to date. Consistent with previous investigations, ML analyses and Bayesian phylogenies using both amino-acid and nucleotide data recover the Euglenophyceae branching most closely to the prasinophyte order Pyramimonadales (Fig. 1, Supplementary Figs S1, S6, S7). As observed in an earlier plDNA phylogenomic study^7^, *Eutreptiella* branches as the earliest lineage within the Euglenophyceae, followed by *Eutreptia*. To test the robustness of the Pyramimonadales + Euglenophyceae relationship (and other relationships described below) using nucleotide data, we also performed ML analyses using different substitution models in addition to the best-fit (GTR) model (Supplementary Figs S2–S4). A Pyramimonadales + Euglenophyceae clade is recovered with strong support in these phylogenies. However, in most cases the pyramimonadalean lineage closest to the Euglenophyceae clade is not well-resolved. In all ML analyses, *Cymbomonas* and *Pyramimonas* group together with good to full support, whereas the branching position of *Pterosperma* differs (Fig. 1, Supplementary Figs S1–S5). Generally, ML bootstrap support for the branching order of *Pterosperma* is low, with the exception of nucleotide analyses using either a JC69 or K80 substitution model: both phylogenies recovered *Pterosperma* branching as sister to *Cymbomonas* and *Pyramimonas* (89% and 90% bootstrap support [BS], respectively), and together this clade formed a sister group to the euglenophytes (95% and 98% BS, respectively). In Bayesian analyses either *Cymbomonas* or *Pyramimonas* branches most closely to the Euglenophyceae (nucleotide vs amino-acid alignments, respectively), but relationships between pyramimonadalean taxa are not well-resolved (Supplementary Figures S6 and S7). This lack of resolution might be due to the limited data available for *Pterosperma*; complete plDNA sequence is lacking, and only 10 genes were included in our analyses. To evaluate whether systematic biases caused by fast-evolving sites could also be contributing to low support, we calculated site-specific substitution rates for our concatenated nucleotide data set and progressively removed the fastest-evolving sites (see methods). As shown in Supplementary Fig. S8 and Table S8, the ML topology described above is recovered in the 95, 90, 85, 80, 75, 70, 65, and 60% datasets, with support for the branching position of *Pterosperma* ranging from 45–63% BS. Overall, the exact pyramimonadalean lineage (or closely related lineage) that was the source of the euglenophyte plastid remains an open question.

Although members of the Euglenophyceae are found in both marine and freshwater environments, it has been suggested that the secondary endosymbiosis event leading to this lineage occurred in a marine habitat^6,41^. This hypothesis is consistent with the marine habitat of early-branching lineages within the extant photosynthetic euglenophytes, *Eutreptiella* and *Eutreptia*^7^, as well as *Rapaza*^44^. The fact that *Pyramimonas* comprises marine species has also been presented as corroborating evidence for a marine origin^41^. However, phylogenetic analyses of the origin of the euglenophyte plastid have so far only included marine pyramimonadalean taxa, whereas many species occur in freshwater environments, including members of the genus *Pyramimonas*. It might still be the case, then, that the euglenophyte plastid is more closely related to freshwater pyramimonadalean lineages. Moreover, the closest known non-photosynthetic relative of the Euglenophyceae, the euglenid lineage *Peranema*^45^, largely comprises freshwater species, suggesting that the host cell involved in secondary endosymbiosis could have been of freshwater origin. Together, these facts leave open the possibility of a freshwater origin of the Euglenophyceae.

**Figure 2:**
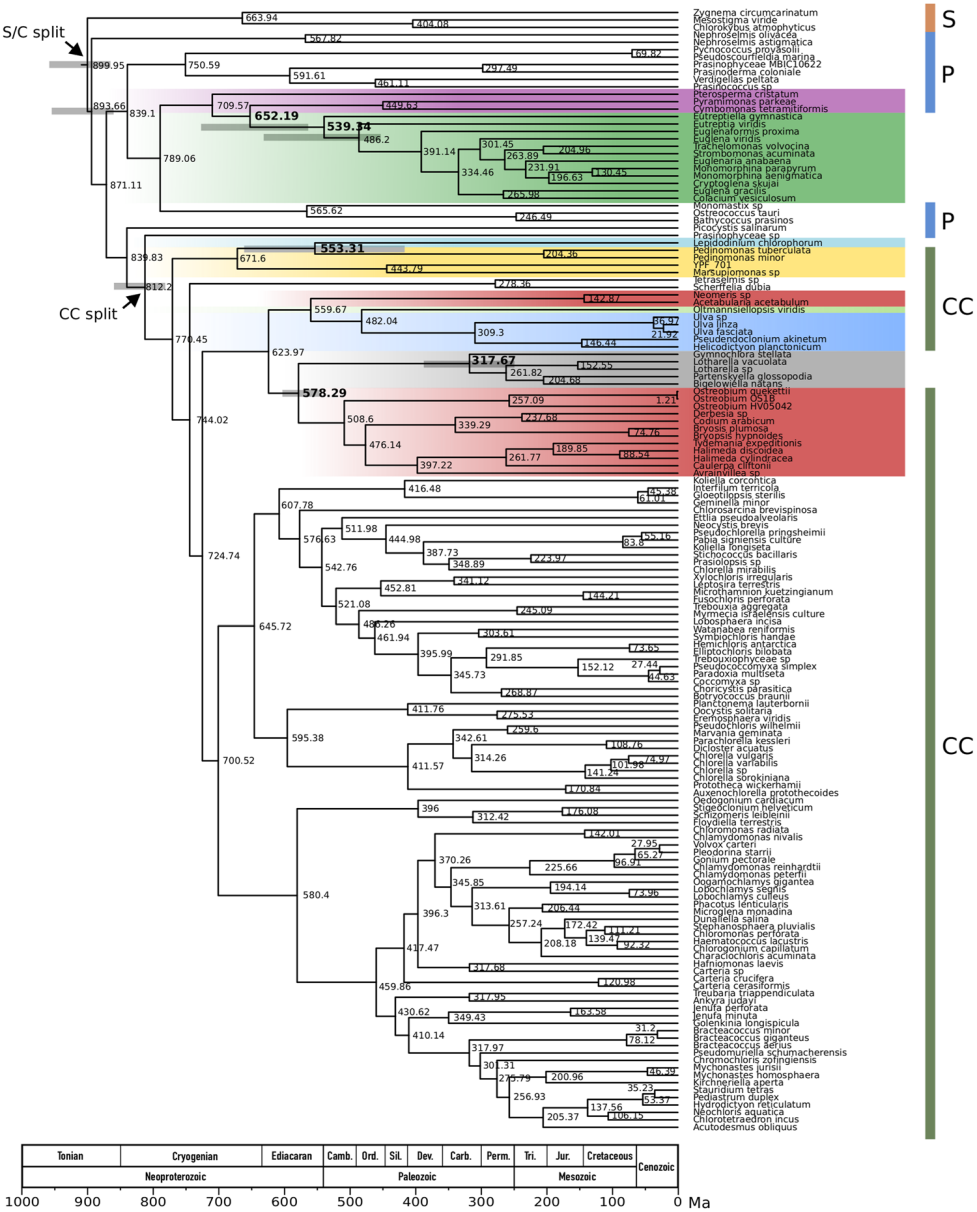
Chronogram of streptophytes, green algae, photosynthetic euglenophytes, the “green” dinoflagellate *Lepidodinium chlorophorum*, and chlorarachniophytes. Node ages were inferred using Bayesian inference assuming a relaxed molecular clock and a set of node age constraints derived from the fossil record as well as node age estimates from previous studies (see Supporting Information File 1). Values at nodes indicate average node ages and grey bars at selected nodes represent 95% confidence intervals. Coloured vertical bars to the right of the phylogeny are labelled: S, Streptophyta; P, prasinophytes; CC, core Chlorophyta. “S/C split” = divergence of the Streptophyta and Chlorophyta. “CC split” = divergence of the prasinophytes and core Chlorophyta. Timeline abbreviations: Camb., Cambrian; Ord., Ordovician; Sil., Silurian; Dev., Devonian; Carb., Carboniferous; Perm., Permian; Tri., Triassic; Jur., Jurassic.

Our relaxed molecular clock analyses indicate that the secondary endosymbiosis event leading to the Euglenophyceae occurred between ~652 and 539 million years ago (Ma) (Fig. 2). The 95% confidence intervals (CI) for these dates overlap (~563–728 and ~453–631 Ma, respectively), and together encompass the late Proterozoic to early Paleozoic. The two timeframe estimates correspond to the split between the Pyramimonadales and the Euglenophyceae plastid lineage, and the start of the radiation of Euglenophyceae, respectively. Thus, the divergence of the euglenophyte plastid lineage may have followed closely on the heels of the initial diversification of marine Pyramimonadales, as recorded by fossil phycomata^46^, biomarker molecular fossils^47^ and molecular clock estimates^48^. However, the uncertain branching position of *Pterosperma* in our phylogenetic analyses means that this date estimate should be interpreted with caution. While we cannot determine at which point in time along the branch leading to Euglenophyceae the secondary endosymbiosis event took place, fossil pellicles indicate that photosynthetic euglenophytes existed at least 450 million years ago^49,50^. Phylogenies of nuclear SSU rDNA recover the photosynthetic marine euglenid *Rapaza viridis* branching as the nearest sister lineage to the remaining photosynthetic euglenophytes^44^. Plastid sequence data from *Rapaza* would therefore be informative in estimating the date of Euglenophyceae radiation, and in narrowing the potential time-frame for the secondary endosymbiosis event. Overall, the time-frame suggested by our analyses is consistent with molecular dating of euglenophyte host cells using nuclear data; Parfrey *et al*.^51^ recovered plastid-containing euglenophytes diverging from a non-photosynthetic bacteriotrophic euglenophyte ~760 Ma, implying that secondary plastid endosymbiosis in the euglenophyte lineage occurred no earlier than this date. Due to our broad time-frame estimate, it is difficult to speculate on global environmental conditions under which secondary endosymbiosis could have taken place; photosynthetic euglenophytes may have originated within freshwater or marine refugia during one of the Cryogenian ice ages or in cold, oligotrophic waters between ice ages.

### Dinoflagellates: Lepidodinium

In the case of the ‘serial secondary’ green dinoflagellate plastid, earlier plastid multi-gene analyses^42^ indicated that the *Lepidodinium* plastid is most closely related to members of the core chlorophytes, with more recent whole-plDNA phylogenomics indicating a sister relationship between *Lepidodinium* and the pedinophyte *Pedinomonas minor*^4^. Pedinophytes are an early-diverging lineage of the core Chlorophyta (CC in Fig. 1)^40^. A caveat of the whole-plDNA study^4^ was that only a single pedinophyte species was included, and so it could not be determined whether the *Lepidodinium* plastid is descended from a pedinophyte or an unknown alga closely related to pedinophytes.

Using additional pedinophyte sequences, our ML analysis of amino-acid data recovers *Lepidodinium* branching within a strongly supported monophyletic pedinophyte clade (91% BS, Fig. 1). Although the branch leading to *Lepidodinium* is very long and potentially leads to issues such as long-branch attraction, this does not appear to be the case here. Our dataset includes four pedinophyte species, representing the broadest taxon sampling to date of this group in phylogenetic analyses of the *Lepidodinium* plastid. Consequently, with the exception of *Lepidodinium*, branches within the pedinophyte clade are of average length compared to other green-algal groups in the phylogeny. Further, a strongly supported *Lepidodinium* + pedinophyte clade is recovered in nucleotide ML phylogenies using the best-fit model (GTR), as well as Bayesian analyses using a site-heterogeneous CATGTR model, which is more robust against LBA artefacts^52^ (Supplementary Figs S1, S6 and S7). In ML analyses of nucleotide data using the much simpler JC69 and K80 models *Lepidodinium* branches as sister to chlorarachniophytes (Supplementary Figs S3–S4), but this is likely due to a poor fit of the model to the data, and/or potentially long-branch attraction given the long branch leading to the chlorarachniophyte clade. Overall we are confident that, consistent with previous investigations, the *Lepidodinium* plastid is indeed most closely related to a pedinophyte lineage. In our analyses *Lepidodinium* branches together with a clade consisting of two *Pedinomonas* species, with *Marsupiomonas* sp. and the newly sequenced strain YPF-701 forming a sister group to the *Lepidodinium*/*Pedinomonas* clade. Hence, our investigation confirms that the *Lepidodinium* plastid did indeed originate from a pedinophyte lineage, rather than a related lineage.

Our molecular clock analysis estimates ~553 Ma for the divergence of the *Lepidodinium* plastid lineage from its pedinophyte sisters, with a 95% CI of ~416–661 Ma (Fig. 2). However, both nuclear SSU and LSU rRNA phylogenies indicate that the *Lepidodinium* dinoflagellate host was a member of the dinoflagellate genus *Gymnodinium*^19,53,54^. The Gymnodiniaceae is nested phylogenetically within a core dinoflagellate clade that began to radiate, according to fossils and molecular clocks, only about 220 million years ago^55–57^. Thus, the incorporation of a plastid into the *Lepidodinium* lineage likely postdates the plastid-lineage divergence time estimated from living taxa by more than 300 million years, possibly occurring as part of the broader radiation of phytoplankton in Mesozoic oceans^8^. As data for only a single *Lepidodinium* species is available we cannot date the radiation of the *Lepidodinium* lineage, and so we lack an estimate for the upper (i.e. most recent) boundary for the timeframe during which endosymbiosis likely occurred. Overall, our results suggest that the *Lepidodinium* plastid lineage is likely to have had an extensive history as a free-living pedinophyte after diverging from other known lineages. It is possible that free-living members are now extinct; alternatively, further sampling of putative pedinophyte diversity might uncover extant members more closely related to the *Lepidodinium* plastid.

### Chlorarachniophytes

Of the three known lineages with secondary green plastids, the ancestor of the chlorarachniophyte plastid has been the most difficult to pin down. The most taxon-rich analysis to date (55 plDNA genes) included five chlorarachniophyte species and recovered a monophyletic chlorarachniophyte clade branching as sister to the order Bryopsidales (three taxa included) with moderate to strong support^5^. The Bryopsidales, together with the order Dasycladales, comprise the siphonous green algae, whose members consist of a giant single filamentous or branched multinucleate cell^58,59^. The recovered tree topology suggested that the ancestor of the chlorarachniophyte plastid might have belonged to Bryopsidales or a closely related group, but could not distinguish between these scenarios due to limited taxon sampling of putative closely related taxa; the Dasycladales were not represented, and the closest relative of the chlorarachniophyte/Bryopsidalean clade was not recovered with statistical support^5^.

In our study we include data from eleven Bryopsidalean taxa from eight families, including several newly sequenced early-branching lineages, providing dense sampling within groups that are closely related to the chlorarachniophyte plastid and allowing us to test the hypothesis that chlorarachniophyte plastids may derive from a lineage within Bryopsidales. Importantly, we also include new data from *Neomeris* sp. and published data for *Acetabularia acetabulum*, members of the order Dasycladales. In some published analyses the Dasycladales group is sister to the Bryopsidales with strong support^40,58,60^, and the inclusion of this lineage in our analyses potentially allows a more-refined resolution of the branching position of the chlorarachniophytes within the green algae.

Our analyses recover chlorarachniophytes branching as sister to the Bryopsidales in all maximum likelihood phylogenies (Fig. 1, Supplementary Figs S1–S5), with bootstrap support for this clade ranging from strong with more sophisticated models (93%, partitioned nucleotide dataset, GTR model) to non-significant using very simple models of sequence evolution (42%, partitioned nucleotide dataset, HKY model). Analyses with our amino-acid dataset recovered the clade with good support (80%, Fig. 1). The consistent recovery of a chlorarachniophyte + Bryopsidales group using different models and data matrices suggests that the relationship is genuine, despite potential long-branch attraction artefacts caused by the extended branch leading to the chlorarachniophyte clade. In Bayesian analyses using a CATGTR model (Supplementary Figs S6–S7), chlorarachniophytes branch within the core Chlorophyta with strong support.

In contrast to the consistent relationship between the chlorarachniophytes and the Bryopsidales, the branching location of the siphonous Dasycladales varied in different analyses. In the best-fit model analysis of nucleotide data (GTR, partitioned), the Dasycladales branched as a sister group to a chlorarachniophyte + Bryopsidales clade with good support (85% BS, Fig. S1), lending support to the hypothesis that the ancestor of the chlorarachniophyte plastid was a lineage of siphonous green algae, rather than a closely related group. The same relationship was recovered using a partitioned HKY model, albeit with no statistical support (17% BS, Supplementary Fig. S2). Analyses with either a partitioned JC69 or K80 model also recovered a Dasycladales + chlorarachniophyte + Bryopsidales clade (66% and 85% BS, respectively), except that *Lepidodinium* branched inside the clade as sister to the chlorarachniophytes (Supplementary Figs S3–S4). Analyses of our amino-acid dataset, on the other hand, recovered Dasycladales grouping as sister to an Oltmannsiellopsidales + Ulvales-Ulotrichales clade with strong support (95% BS; Fig. 1). This relationship was also recovered with strong support in Bayesian analyses of both nucleotide and amino-acid data (Bayesian Posterior Probability of 1; Supplementary Figs S6 and S7). Notably, a weak association between Dasycladales and an Oltmannsiellopsidales + Ulvales–Ulotrichales clade has been reported previously^61^, although other analyses recover a sister relationship between the Dasycladales and Trentepohliales^40,62^. To test whether the branching positions of the Dasycladales and Bryopsidales were affected by inclusion of chlorarachniophyte sequences, we performed a partitioned ML analysis of both nucleotide and amino-acid data with all secondary plastid sequences removed (Supplementary Figs S9 and S10). In both cases the same respective topologies were recovered for these groups: a Dasycladales + Bryopsidales clade received 97% BS in the nucleotide analysis, and a Dasycladales + Oltmannsiellopsidales + Ulvales-Ulotrichales clade received 98% BS in the amino-acid analysis, suggesting that the secondary plastid sequences are not affecting these phylogenetic relationships. Finally, to examine whether inclusion of some very recently-released ulvophycean plDNAs^63^ affected the topologies recovered with our datasets, we performed maximum likelihood analyses with more early-branching Ulvophyceae lineages (Dataset B, Supplementary Table S9; see highlighted taxa). The position of chlorachniophyte plastids in these phylogenies was entirely consistent with analyses above (Supplementary Figs S11 and S12). Further, analysis of the amino-acid alignment from Dataset B using a more sophisticated site-heterogeneous LG + G4 + C60 + F + PMSF model^36^, which estimates site-specific amino-acid profiles based on C60 empirical frequency profiles^64^, also recovered a chlorarachniophyte + Bryopsidales clade (73% BS), with a Dasycladales + Oltmannsiellopsidales + Ignatiales + Ulvales-Ulotrichales clade receiving 99% BS (Supplementary Fig. S13).

To investigate possible reasons behind these two well-resolved but conflicting topologies we carried out a series of analyses, as described in Supplementary Information File S1. Briefly, we calculated site-wise support for the conflicting topologies from our alignments, and showed that the strong support for a Dasycladales + chlorarachniophyte + Bryopsidales clade in our nucleotide matrix largely originated from third-codon positions. Typically, the rate of nucleotide substitution is higher at third codon positions^65,66^, leading to substitutional saturation and erosion of accurate phylogenetic signal. Therefore, removal of third codon positions for protein-coding genes is a commonly employed strategy. Given this reasoning and the overall balance of evidence from our analyses (Supplementary Information File S1, Supplementary Fig. S5), we suggest that the Dasycladales + Oltmannsiellopsidales + Ulvales-Ulotrichales clade represents the most likely species relationship hypothesis based on plastid genome data, and that this clade might be sister to the chlorarachniophyte + Bryopsidales group (Fig. 1, Supplementary Fig. S5). This topology is broadly consistent with that recovered by Suzuki *et al*.^5^, although in that study the chlorodendrophyceaen *Tetraselmis* branched inside the Ulvophycean + chlorarachniophyte clade as sister to the Ulvales-Ulotrichales group, and the sister relationship between the Ulvales-Ulotrichales and the Chlorarachniophyte + Bryopsidales groups was not recovered with statistical support.

Overall, our phylogenetic results leave open several possibilities for the origin of chlorarachniophyte plastids. If the relationship recovered in Fig. 1 is correct, they could be derived from a single-celled ulvophycean lineage, and a siphonous physiology could have evolved independently in the lineages leading to the Dasycladales and Bryopsidales following this secondary endosymbiosis event. Alternatively, the Dasycladales + Bryopsidales grouping observed in our nucleotide analyses could represent the correct species relationship. Notably, phylogenies derived from mostly nuclear data^60^ also recover a strongly supported Dasycladales + Bryopsidales clade. If this relationship is indeed correct, it implies that the ancestor of the chlorarachniophyte plastid was a lineage of siphonous green algae.

Phylogenetic analyses suggest that the host cell that gave rise to the chlorarachniophytes was a Cercozoan amoeba^67,68^, likely similar to the small marine predator *Minorisa minuta*^69^. Cercozoans are an extremely diverse phylum of protists within the supergroup Rhizaria, and include gliding zooflagellates, filose amoebae and plasmodiophorid plant parasites^68^. It is difficult to envisage how secondary endosymbiosis involving a cercozoan host cell and a green seaweed could take place. Bryopsidales are siphonous macroalgae that are often branched and have multinucleate cells with many plastids. However, two observations suggest a possible mechanism. Firstly, amoeboid life stages of the chlorarachniophyte *Cryptochlora perforans* have been found to be chemotactically attracted to damaged algal filaments of the Bryopsidalean alga *Boodleopsis pusilla*, and subsequently perforate and penetrate such filaments and eat part of their contents^70^. Secondly, a unique and extraordinary wound response has been observed in bryopsidalean seaweeds: in cells of *Bryopsis plumosa*, protoplasm is extruded from injured cells, cell organelles aggregate, protoplasts are formed with a new cell membrane, and new cell walls are produced^71^. One can therefore envisage a cercozoan-like host cell feeding on siphonous algal protoplasts, seemingly providing an excellent opportunity for secondary endosymbiosis to take place. Alternatively, endosymbiosis in this lineage could have involved a cercozoan host capturing a microscopic precursor species to siphonous green algae (cf. Cocquyt *et al*.^60^). If the chlorachniophyte plastid is indeed derived from a lineage of siphonous greens, it is probable that the endosymbiotic event took place in a near-shore benthic marine environment, as all extant siphonous green algae are marine (with one derived exception), as are the closest known non-photosynthetic extant relatives of chlorarachniophytes^69^.

Relaxed molecular clock analyses suggest that the green algal lineage leading to the chlorarachniophyte plastid diverged from the Bryopsidales ~578 Ma (95% CI ~546–603), indicating that secondary endosymbiosis occurred after this time (Fig. 2). The date of radiation of extant sampled chlorarachniophytes (which must have occurred after plastid acquisition) was estimated at ~318 Ma (95% CI ~250–388). Thus, our analyses suggest that endosymbiosis occurred at some point between 578–318 Ma. The older boundary of this timeframe is consistent with the late Proterozoic and early Paleozoic radiation of the siphonous green algae with which chlorarachniophyte plastids may be associated, as recorded by the skeletal remains and occasional organic compressions of the former lineages^58,72^. Moreover, it is consistent with molecular clock analyses estimated from nuclear genes, which recover the chlorarachniophyte *Bigelowiella* diverging from other cercozoans around 1,000 Ma^51^. While additional sampling of putative later-branching cercozoans that are closely related to the chlorarachniophyte host lineage could bring this time estimate forward, overall this window (1,000–578 Ma) is consistent with suggestions of a putative cryptic endosymbiosis of a red algal plastid-containing eukaryote in the common ancestor of extant chlorarachniophytes, prior to acquisition of the green-algal plastid^73^.

## Conclusions

Here we present advances in pinpointing the origin of secondary green plastids in the three lineages known to contain them. We improve the density of taxon sampling in green algal lineages closely related to secondary plastids, using new plastid genomes presented in this study along with new publically available data, allowing a more precise understanding of these relationships. We show that the *Lepidodinium* plastid originated from a pedinophyte rather than a closely related lineage. It will be interesting to see whether future discoveries of pedinophyte diversity can identify extant free-living members more closely related to the *Lepidodinium* plastid. While the euglenophyte plastid appears likely to have originated from a prasinophyte within the order Pyramimonadales, we cannot rule out an unknown closely related lineage. For chlorarachniophytes, our analyses suggest that the secondary plastid originated from a precursor of siphonous green algae or a closely related unknown lineage.

Nearly all known ulvophyte lineages were sampled here, and the main un-sampled lineage has highly deviant chloroplast genomes^74^, so further clarification does not seem likely unless as-yet-unknown lineages are discovered. Moreover, overall placement of the chlorarachniophyte plastid within the Ulvophyceae and the core Chlorophyta more broadly is hampered by weak signal in chloroplast genome datasets, which leads to poorly resolved or incongruent relationships between the main clades of core Chlorophyta^66^. Phylogenetic studies using nuclear endosymbiont-derived genes may further resolve the origin of the chlorarachniophyte plastid, but very little nuclear data is currently available. Finally, we integrate our phylogenies with fossil information to present estimates of timeframes during which secondary plastid endosymbiosis is likely to have occurred for each of the three secondary lineages, using a relaxed molecular clock model to better account for the changes in molecular evolutionary rates during secondary endosymbiosis. On balance, our results suggest that the secondary endosymbiosis event leading to *Lepidodinium* occurred more recently than endosymbiosis events leading to the euglenophyte and chlorarachniophyte lineages.

## Electronic supplementary material

Supplementary Figures and Information

Supplementary Tables S2 - S9
