# Supplementary material for: Plastid phylogenomics with broad taxon sampling further elucidates the distinct evolutionary origins and timing of secondary green plastids
Source: Sci Rep. 2018 Jan 24;8:1523. doi: 10.1038/s41598-017-18805-w (PMC5784168; doi:10.1038/s41598-017-18805-w)
Supplement: Supplementary file 1 — Supplementary Figures and Information [file 41598_2017_18805_MOESM1_ESM.pdf]

Supplementary Information

Plastid phylogenomics with broad taxon sampling further elucidates the distinct evolutionary origins and timing of secondary green plastids.

Chris Jackson, Andrew H. Knoll, Cheong Xin Chan, Heroen Verbruggen.

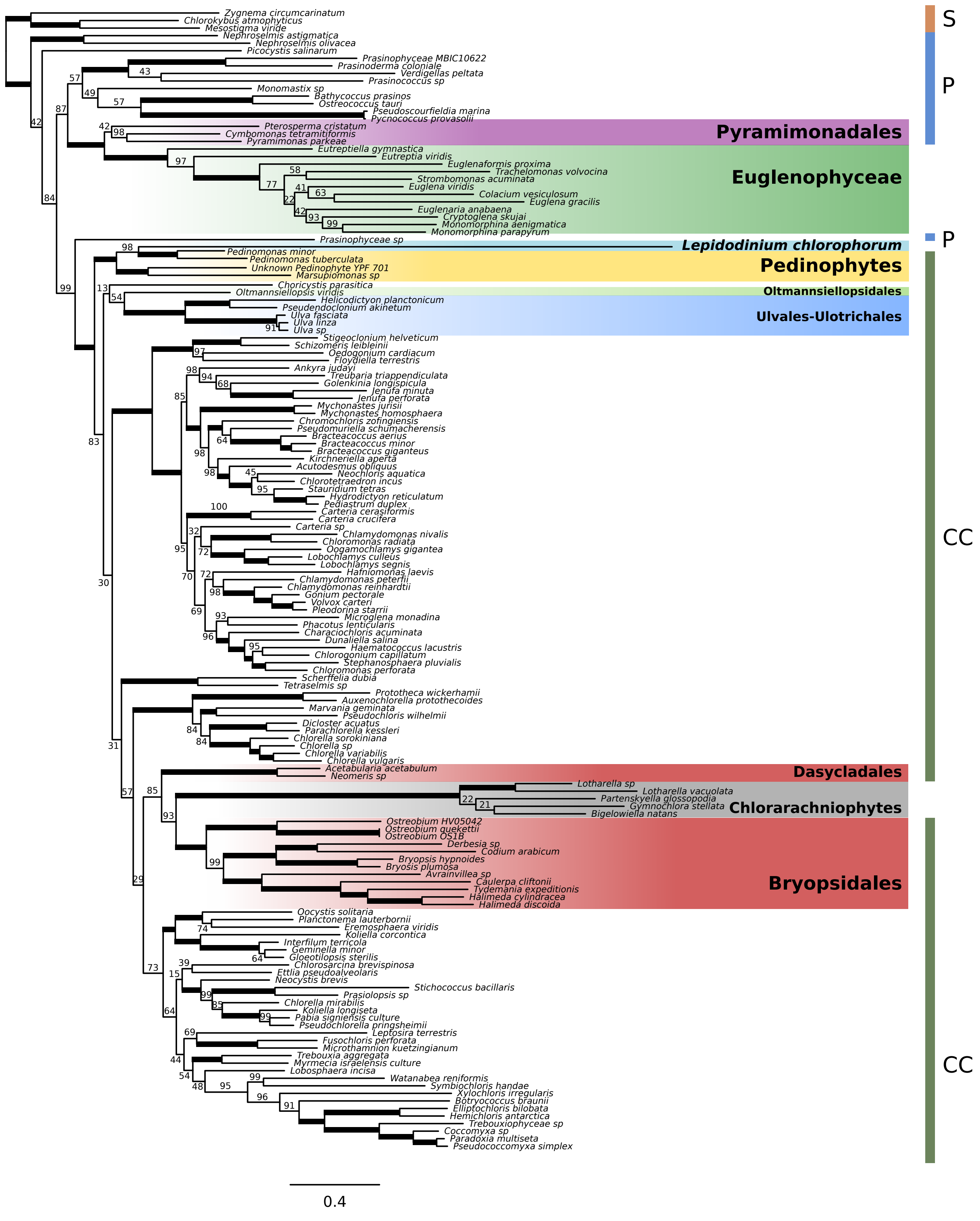

Figure S1. RAxML phylogenetic analysis (GTR model, partitioned by gene and codon position via PartitionFinder) inferred from a nucleotide alignment of 64 plastid genes from streptophytes, green algae, photosynthetic euglenophytes, the "green" dinoflagellate *Lepidodinium chlorophorum*, and chlorarachniophytes. Thick branches have full ML bootstrap support. Coloured vertical bars to the right of the phylogeny are labelled: S, Streptophytes; P, prasinophytes; CC, core Chlorophyta. Branch lengths are proportional to the number of substitution per site.

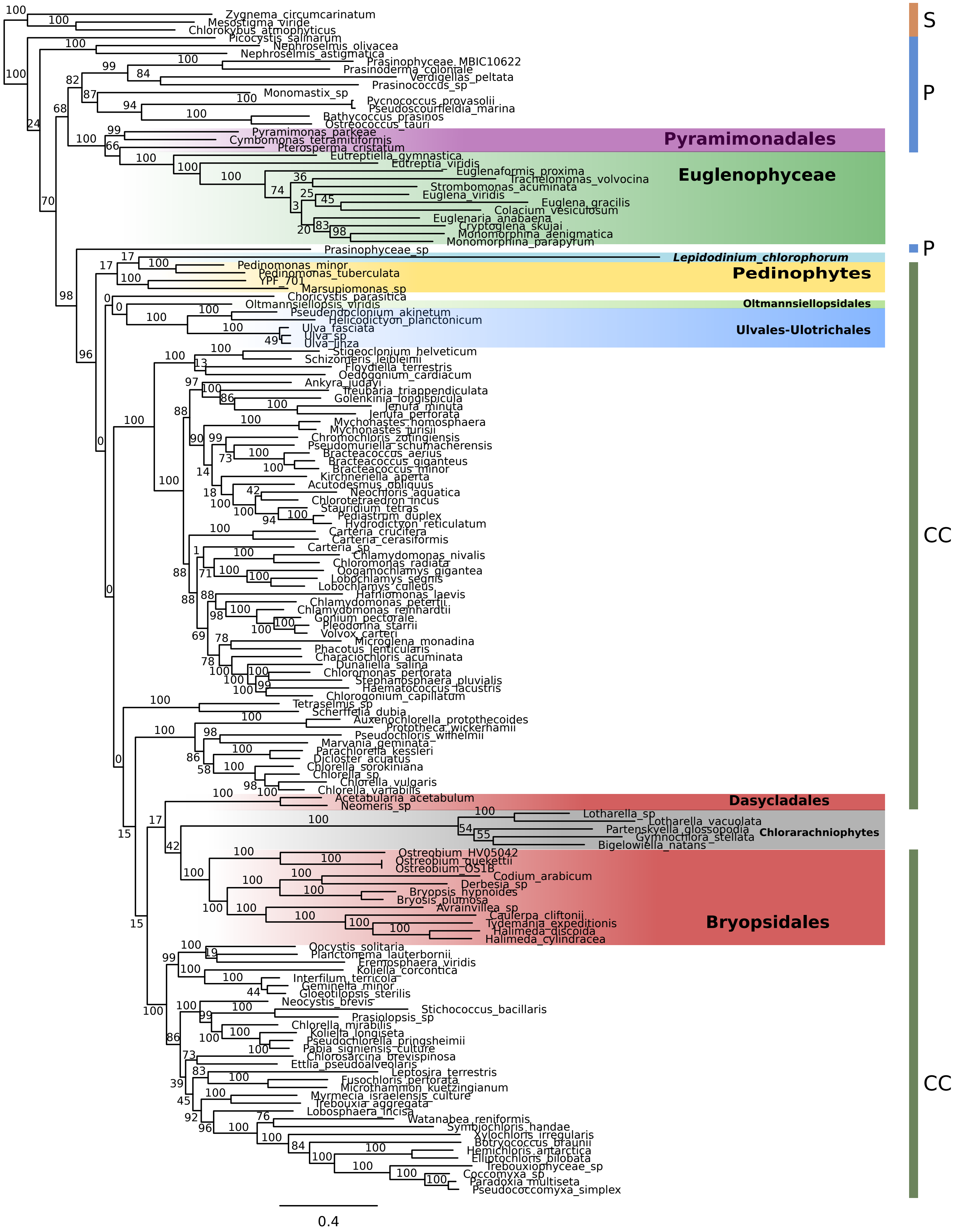

Figure S2. RAxML phylogenetic analysis (HKY model, partitioned by gene and codon position via PartitionFinder) inferred from a nucleotide alignment of 64 plastid genes from streptophytes, green algae, photosynthetic euglenophytes, the “green” dinoflagellate *Lepidodinium chlorophorum*, and chlorarachniophytes. Coloured vertical bars to the right of the phylogeny are labelled: S, Streptophytes; P, prasinophytes; CC, core Chlorophyta. Branch lengths are proportional to the number of substitution per site.

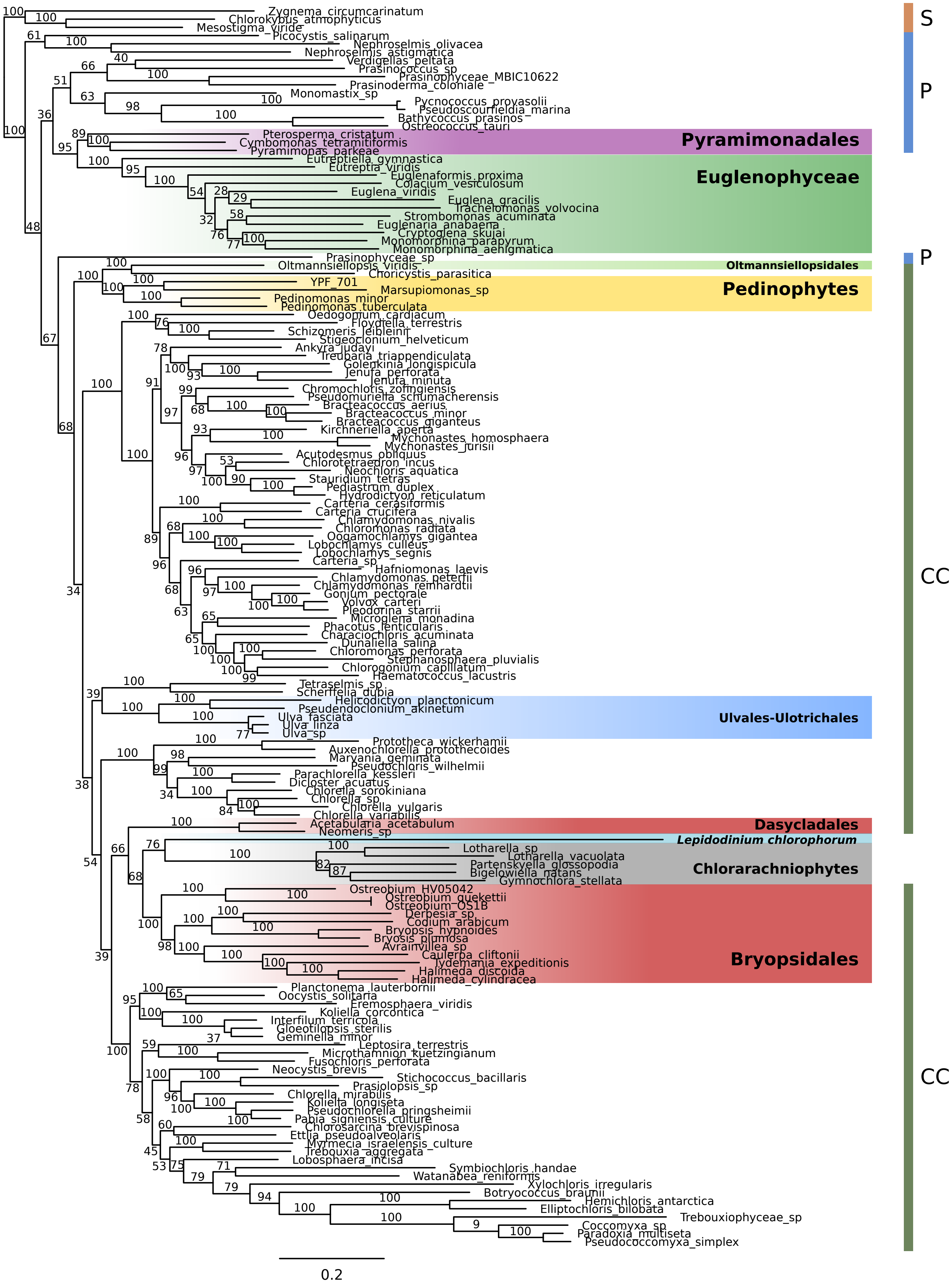

Figure S3. RAxML phylogenetic analysis (JC69 model, partitioned by gene and codon position via PartitionFinder) inferred from a nucleotide alignment of 64 plastid genes from streptophytes, green algae, photosynthetic euglenophytes, the "green" dinoflagellate *Lepidodinium chlorophorum*, and chlorarachniophytes. Coloured vertical bars to the right of the phylogeny are labelled: S, Streptophytes; P, prasinophytes; CC, core Chlorophyta. Branch lengths are proportional to the number of substitution per site.

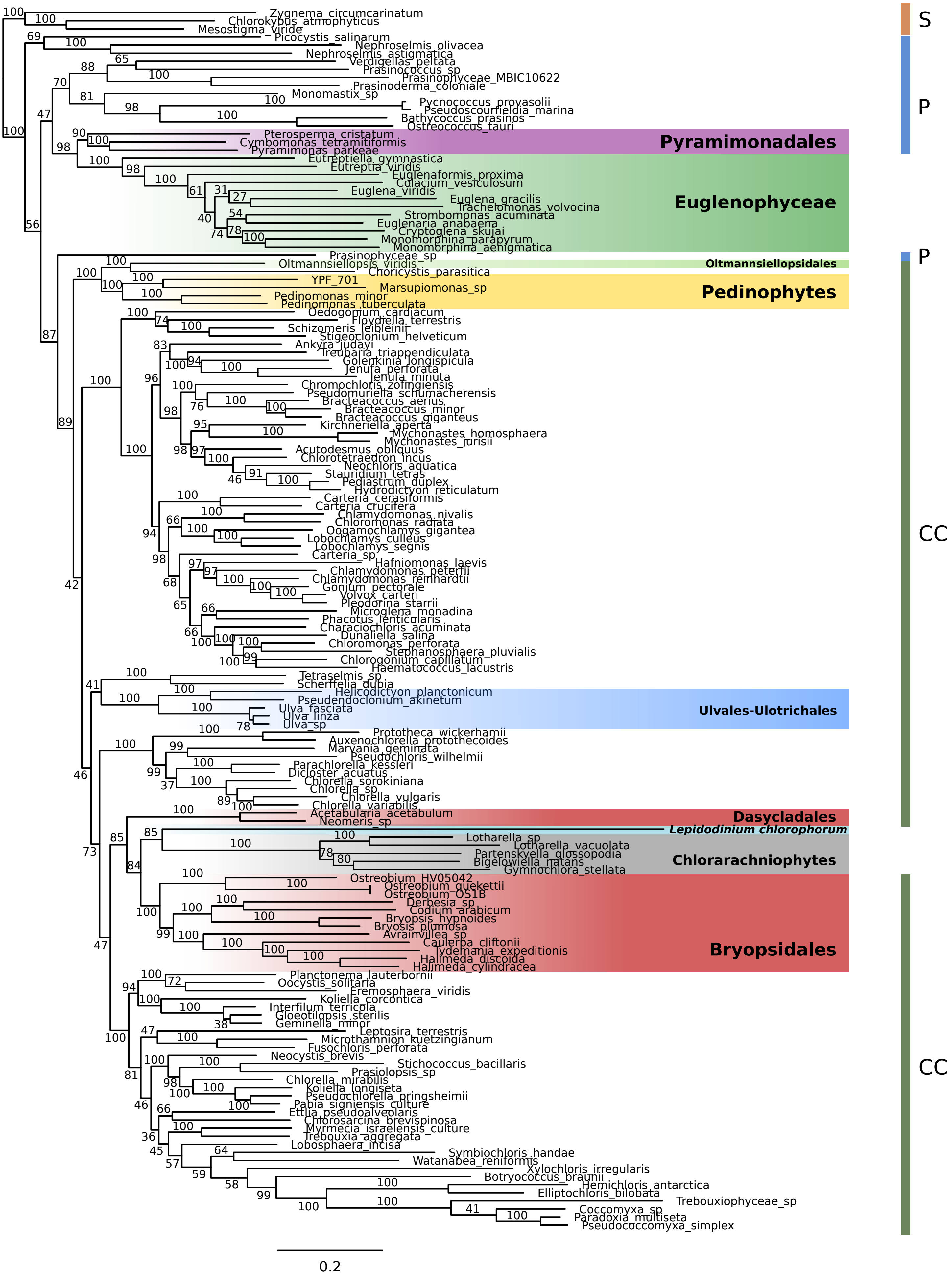

Figure S4. RAxML phylogenetic analysis (K80 model, partitioned by gene and codon position via PartitionFinder) inferred from a nucleotide alignment of 64 plastid genes from streptophytes, green algae, photosynthetic euglenophytes, the “green” dinoflagellate *Lepidodinium chlorophorum*, and chlorarachniophytes. Coloured vertical bars to the right of the phylogeny are labelled: S, Streptophytes; P, prasinophytes; CC, core Chlorophyta. Branch lengths are proportional to the number of substitution per site.

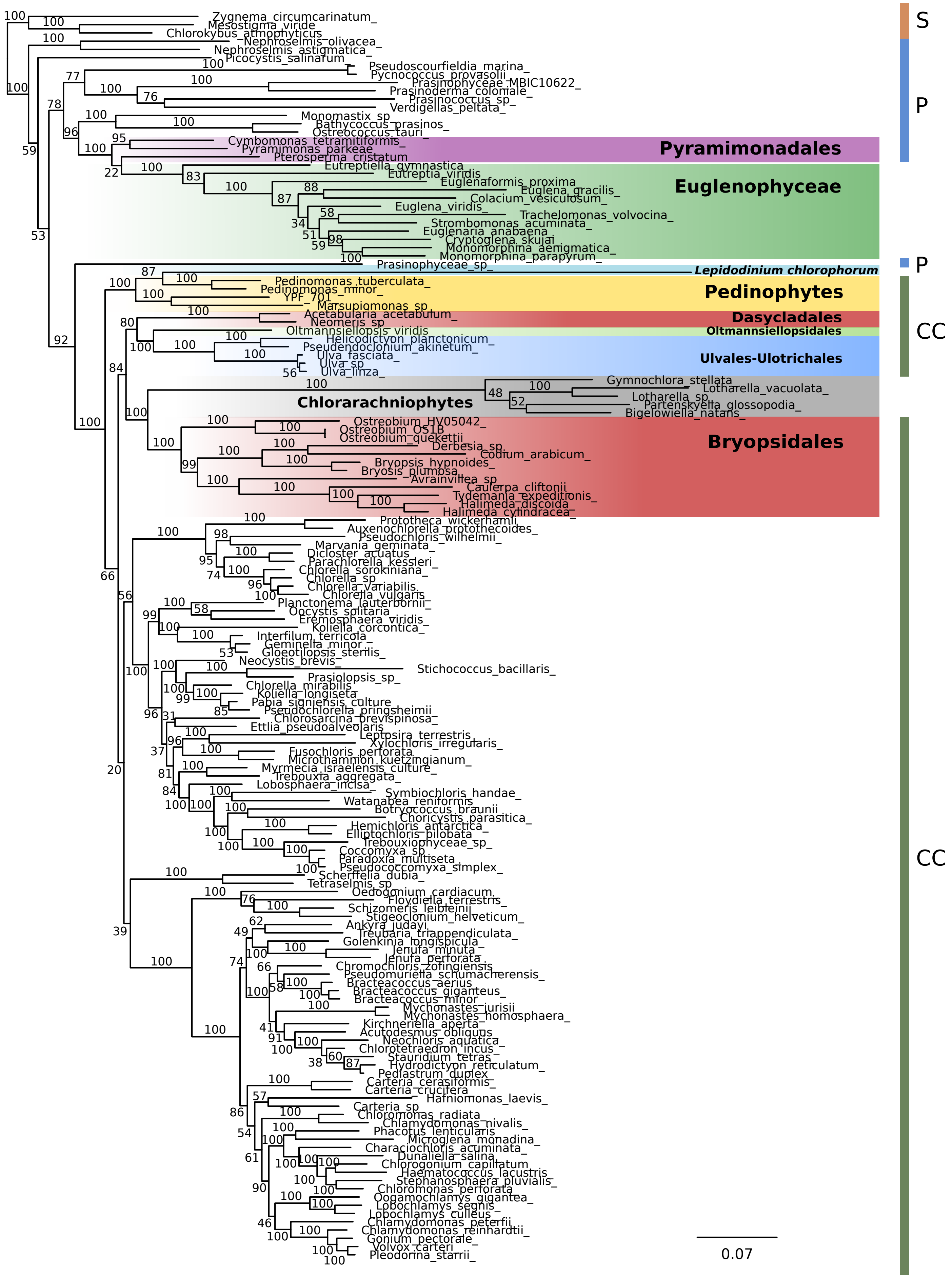

Figure S5. RAxML phylogenetic analysis (GTR model, partitioned by codon position) inferred from a nucleotide alignment of 64 plastid genes from streptophytes, green algae, photosynthetic euglenophytes, the "green" dinoflagellate *Lepidodinium chlorophorum*, and chlorarachniophytes. Third codon-positions were removed from the supermatrix. Coloured vertical bars to the right of the phylogeny are labelled: S, Streptophytes; P, prasinophytes; CC, core Chlorophyta. Branch lengths are proportional to the number of substitution per site.

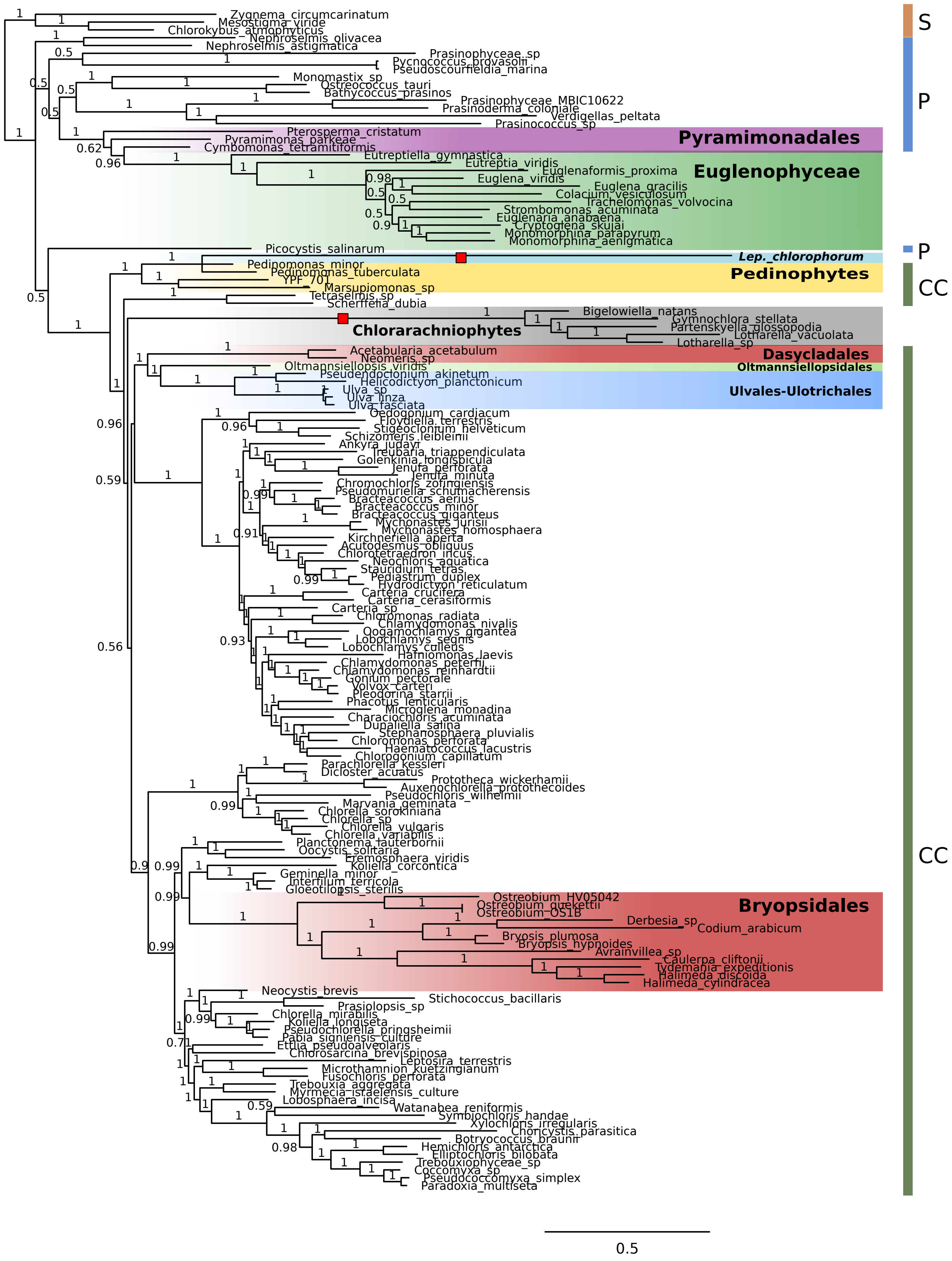

Figure S6. Bayesian phylogenetic analysis (CATGTR model) inferred from a nucleotide alignment of 64 plastid genes from streptophytes, green algae, photosynthetic euglenophytes, the “green” dinoflagellate *Lepidodinium chlorophorum*, and chlorarachniophytes. Branch lengths are proportional to the number of substitution per site. Branches with red squares are drawn at 50% actual length for illustrative purposes.

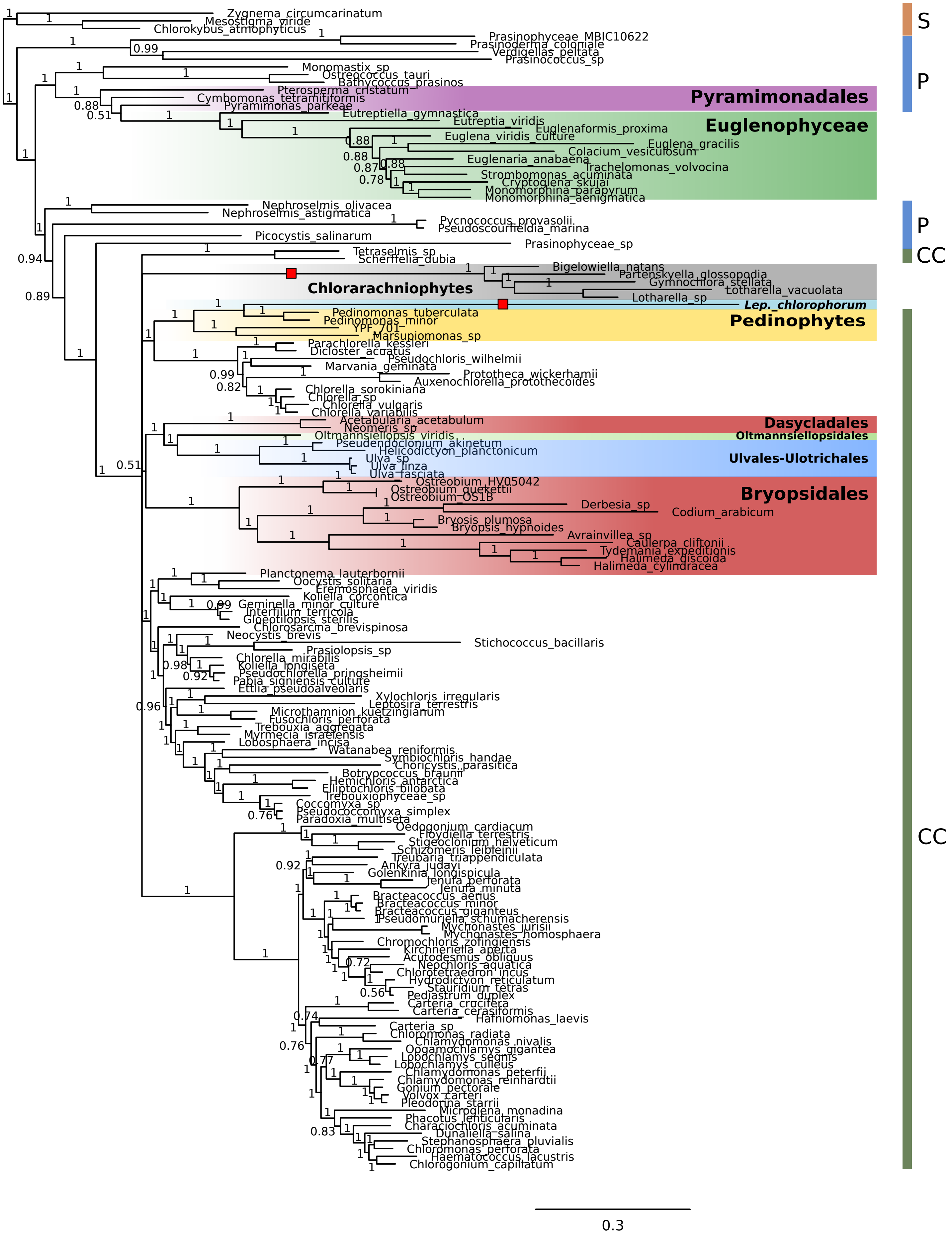

Figure S7. Bayesian phylogenetic analysis (CATGTR model) inferred from an amino acid alignment of 64 plastid genes from streptophytes, green algae, photosynthetic euglenophytes, the "green" dinoflagellate *Lepidodinium chlorophorum*, and chlorarachniophytes. Branch lengths are proportional to the number of substitution per site. Branches with red squares are drawn at 50% actual length for illustrative purposes.

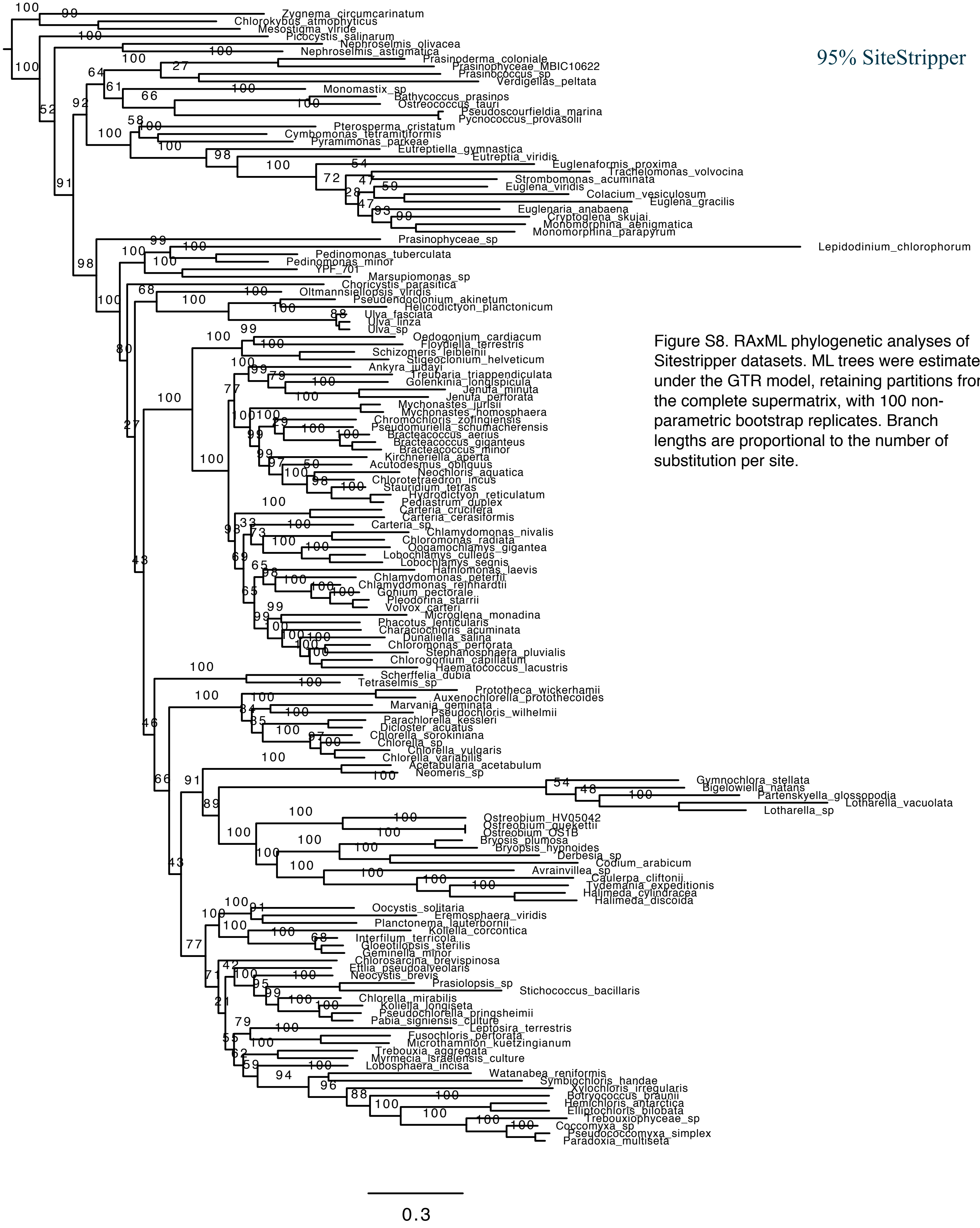

Figure S8. RAxML phylogenetic analyses of Sitestripper datasets. ML trees were estimated under the GTR model, retaining partitions from the complete supermatrix, with 100 non-parametric bootstrap replicates. Branch lengths are proportional to the number of substitution per site.

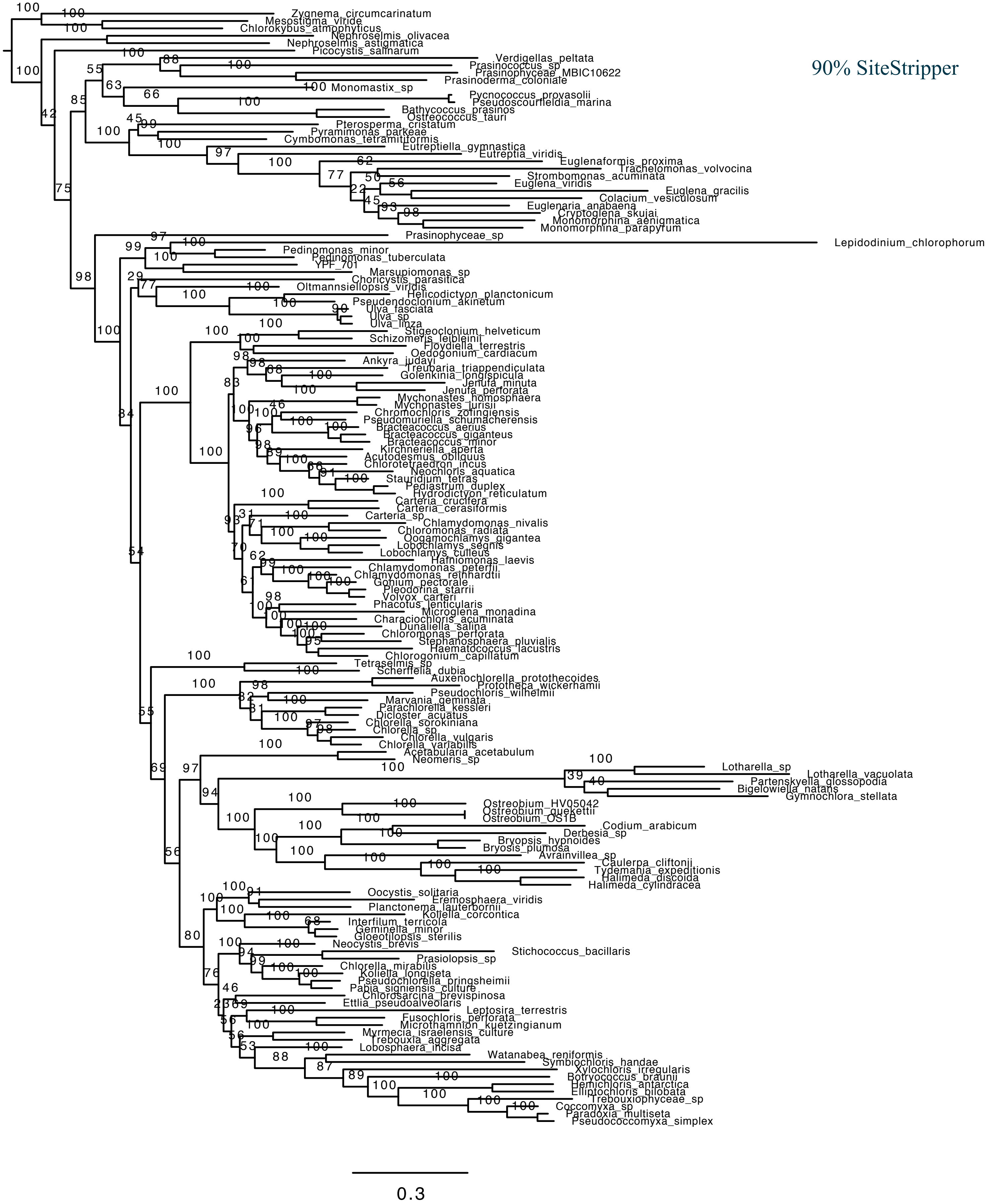
90% SiteStripper
Zygnema_circumcarinatum
Mesostigma_viride
Chlorokybus_atmophyticus
Nephroselmis_olivacea
Nephroselmis_astigmatica
Picocystis_salinarum
Verdigellas_peltata
Prasinococcus_sp
Prasinophyceae_MBIC10622
Prasinoderma_coloniale
Monomastix_sp
Pycnococcus_provasolii
Pseudoscourfieldia_marina
Bathycoccus_prasinos
Ostreococcus_tauri
Pterosperma_cristatum
Pyramimonas_parkeae
Cymbomonas_tetramitiformis
Eutreptiella_gymnastica
Eutreptia_viridis
Euglenaformis_proxima
Trachelomonas_volvocina
Strombomonas_acuminata
Euglena_viridis
Euglena_gracilis
Colacium_vesiculosum
Euglenaria_anabaena
Cryptoglena_skujai
Monomorphina_aenigmatica
Monomorphina_parapyrum
Prasinophyceae_sp
Lepidodinium_chlorophorum
Pedinomonas_minor
Pedinomonas_tuberculata
YPF_701
Marsupiomonas_sp
Choricystis_parasitica
Oltmannsiellopsis_viridis
Helicodictyon_planctonicum
Pseudendoclonium_akinetum
Ulva_fasciata
Ulva_sp
Ulva_linza
Stigeoclonium_helveticum
Schizomeris_leibleinii
Floydiella_terrestris
Oedogonium_cardiacum
Ankyra_judayi
Treubaria_triappendiculata
Golenkinia_longispicula
Jenufa_minuta
Jenufa_perforata
Mychonastes_homosphaera
Mychonastes_jurisii
Chromochloris_zofingiensis
Pseudomuriella_schumacherensis
Bracteacoccus_aerius
Bracteacoccus_giganteus
Bracteacoccus_minor
Kirchneriella_aperta
Acutodesmus_obliquus
Chlorotetraedron_incus
Neochloris_aquatica
Stauridium_tetras
Pediastrum_duplex
Hydrodictyon_reticulatum
Carteria_crucifera
Carteria_cerasiformis
Carteria_sp
Chlamydomonas_nivalis
Chloromonas_radiata
Oogamochlamys_gigantea
Lobochlamys_segnis
Lobochlamys_culleus
Hafniomonas_laevis
Chlamydomonas_peterfii
Chlamydomonas_reinhardtii
Gonium_pectorale
Pleodorina_starrii
Volvox_carteri
Phacotus_lenticularis
Microglena_monadina
Characiochloris_acuminata
Dunaliella_salina
Chloromonas_perforata
Stephanosphaera_pluvialis
Haematococcus_lacustris
Chlorogonium_capillatum
Tetraselmis_sp
Scherffelia_dubia
Auxenochlorella_protothecoides
Prototheca_wickerhamii
Pseudochloris_wilhelmii
Marvania_geminata
Parachlorella_kessleri
Dicloster_acuatus
Chlorella_sorokiniana
Chlorella_sp
Chlorella_vulgaris
Chlorella_variabilis
Acetabularia_acetabulum
Neomeris_sp
Lotharella_sp
Lotharella_vacuolata
Partenskyella_glossopodia
Bigelowiella_natans
Gymnochlora_stellata
Ostreobium_HV05042
Ostreobium_quekettii
Ostreobium_OS1B
Codium_arabicum
Derbesia_sp
Bryopsis_hypnoides
Bryosis_plumosa
Avrainvillea_sp
Caulerpa_cliftonii
Tydemania_expeditionis
Halimeda_discoida
Halimeda_cylindracea
Oocystis_solitaria
Eremosphaera_viridis
Planctonema_lauterbornii
Koliella_corcontica
Interfilum_terricola
Geminella_minor
Gloeotilopsis_sterilis
Neocystis_brevis
Stichococcus_bacillaris
Prasiolopsis_sp
Chlorella_mirabilis
Koliella_longiseta
Pseudochlorella_pringsheimii
Pabia_signiensis_culture
Chlorosarcina_brevispinosa
Ettlia_pseudoalveolaris
Leptosira_terrestris
Fusochloris_perforata
Microthamnion_kuetzingianum
Myrmecia_israelensis_culture
Trebouxia_aggregata
Lobosphaera_incisa
Watanabea_reniformis
Symbiochloris_handae
Xylochloris_irregularis
Botryococcus_braunii
Hemichloris_antarctica
Elliptochloris_bilobata
Trebouxiophyceae_sp
Coccomyxa_sp
Paradoxia_multiseta
Pseudococcomyxa_simplex
0.3

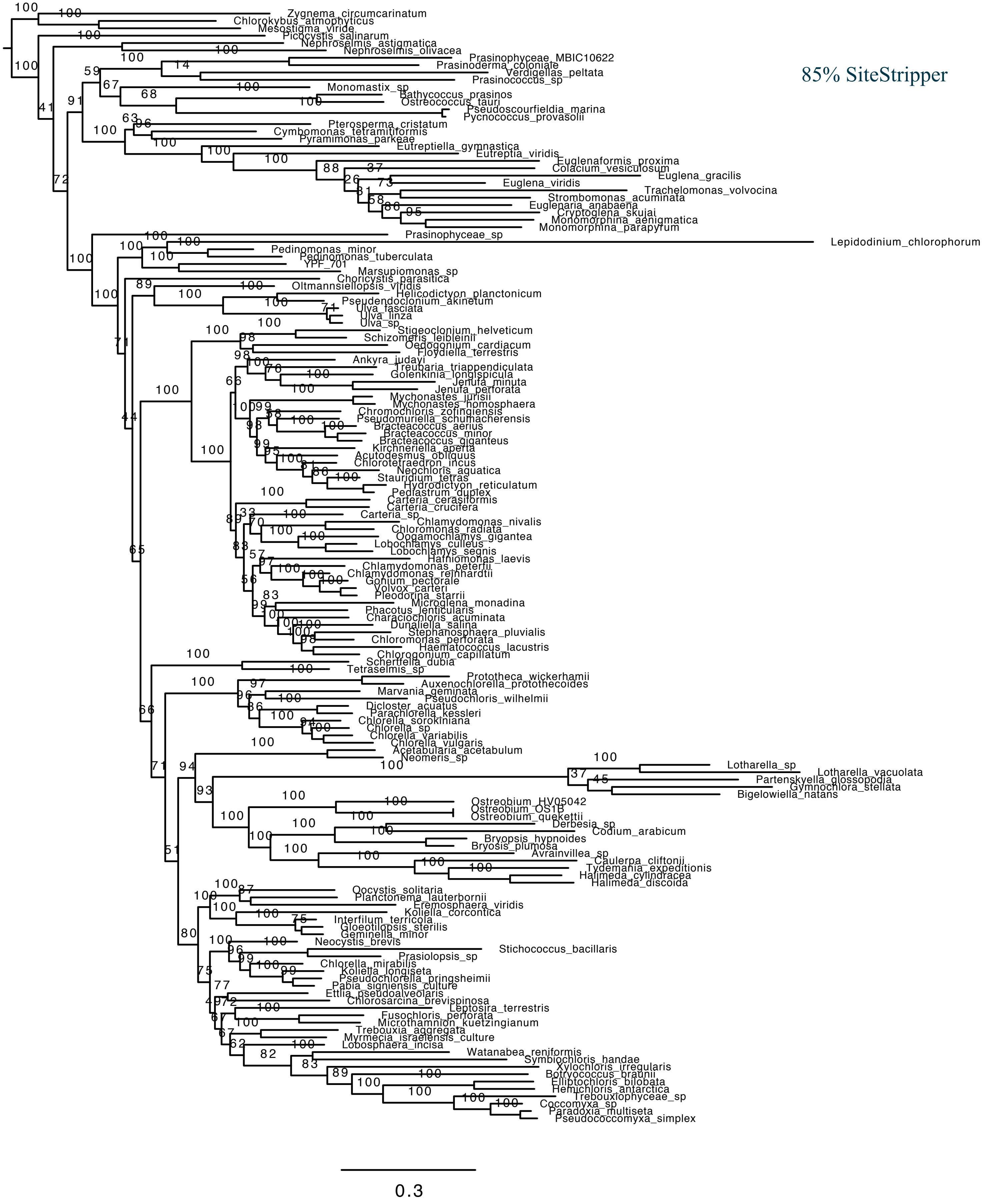
85% SiteStripper
Zygnema_circumcarinatum
Chlorokybus_atmophyticus
Mesostigma_viride
Picocystis_salinarum
Nephroselmis_astigmatica
Nephroselmis_olivacea
Prasinophyceae_MBIC10622
Prasinoderma_coloniale
Verdigellas_peltata
Prasinococcus_sp
Monomastix_sp
Bathycoccus_prasinos
Ostreococcus_tauri
Pseudoscourfieldia_marina
Pycnococcus_provasolii
Pterosperma_cristatum
Cymbomonas_tetramitiformis
Pyramimonas_parkeae
Eutreptiella_gymnastica
Eutreptia_viridis
Euglenaformis_proxima
Colacium_vesiculosum
Euglena_gracilis
Euglena_viridis
Trachelomonas_volvocina
Strombomonas_acuminata
Euglenaria_anabaena
Cryptoglena_skujai
Monomorphina_aenigmatica
Monomorphina_parapyrum
Prasinophyceae_sp
Lepidodinium_chlorophorum
Pedinomonas_minor
Pedinomonas_tuberculata
YPF_701
Marsupiomonas_sp
Choricystis_parasitica
Oltmannsiellopsis_viridis
Helicodictyon_planctonicum
Pseudendoclonium_akinetum
Ulva_fasciata
Ulva_linza
Ulva_sp
Stigeoclonium_helveticum
Schizomeris_leibleinii
Oedogonium_cardiacum
Floydiella_terrestris
Ankyra_judayi
Treubaria_triappendiculata
Golenkinia_longispicula
Jenufa_minuta
Jenufa_perforata
Mychonastes_jurisii
Mychonastes_homosphaera
Chromochloris_zofingiensis
Pseudomuriella_schumacherensis
Bracteacoccus_aerius
Bracteacoccus_minor
Bracteacoccus_giganteus
Kirchneriella_aperta
Acutodesmus_obliquus
Chlorotetraedron_incus
Neochloris_aquatica
Stauridium_tetras
Hydrodictyon_reticulatum
Pediastrum_duplex
Carteria_cerasiformis
Carteria_crucifera
Carteria_sp
Chlamydomonas_nivalis
Chloromonas_radiata
Oogamochlamys_gigantea
Lobochlamys_culleus
Lobochlamys_segnis
Hafniomonas_laevis
Chlamydomonas_peterfii
Chlamydomonas_reinhardtii
Gonium_pectorale
Volvox_carteri
Pleodorina_starrii
Microglena_monadina
Phacotus_lenticularis
Characiochloris_acuminata
Dunaliella_salina
Stephanosphaera_pluvialis
Chloromonas_perforata
Haematococcus_lacustris
Chlorogonium_capillatum
Scherffelia_dubia
Tetraselmis_sp
Prototheca_wickerhamii
Auxenochlorella_protothecoides
Marvania_geminata
Pseudochloris_wilhelmii
Dicloster_acuatus
Parachlorella_kessleri
Chlorella_sorokiniana
Chlorella_sp
Chlorella_variabilis
Chlorella_vulgaris
Acetabularia_acetabulum
Neomeris_sp
Lotharella_sp
Lotharella_vacuolata
Partenskyella_glossopodia
Gymnochlora_stellata
Bigelowiella_natans
Ostreobium_HV05042
Ostreobium_OS1B
Ostreobium_quekettii
Derbesia_sp
Codium_arabicum
Bryopsis_hypnoides
Bryosis_plumosa
Avrainvillea_sp
Caulerpa_cliftonii
Tydemania_expeditionis
Halimeda_cylindracea
Halimeda_discoida
Oocystis_solitaria
Planctonema_lauterbornii
Eremosphaera_viridis
Koliella_corcontica
Interfilum_terricola
Gloeotilopsis_sterilis
Geminella_minor
Neocystis_brevis
Stichococcus_bacillaris
Prasiolopsis_sp
Chlorella_mirabilis
Koliella_longiseta
Pseudochlorella_pringsheimii
Pabia_signiensis_culture
Ettlia_pseudoalveolaris
Chlorosarcina_brevispinosa
Leptosira_terrestris
Fusochloris_perforata
Microthamnion_kuetzingianum
Trebouxia_aggregata
Myrmecia_israelensis_culture
Lobosphaera_incisa
Watanabea_reniformis
Symbiochloris_handae
Xylochloris_irregularis
Botryococcus_braunii
Elliptochloris_bilobata
Hemichloris_antarctica
Trebouxiophyceae_sp
Coccomyxa_sp
Paradoxia_multiseta
Pseudococcomyxa_simplex
0.3

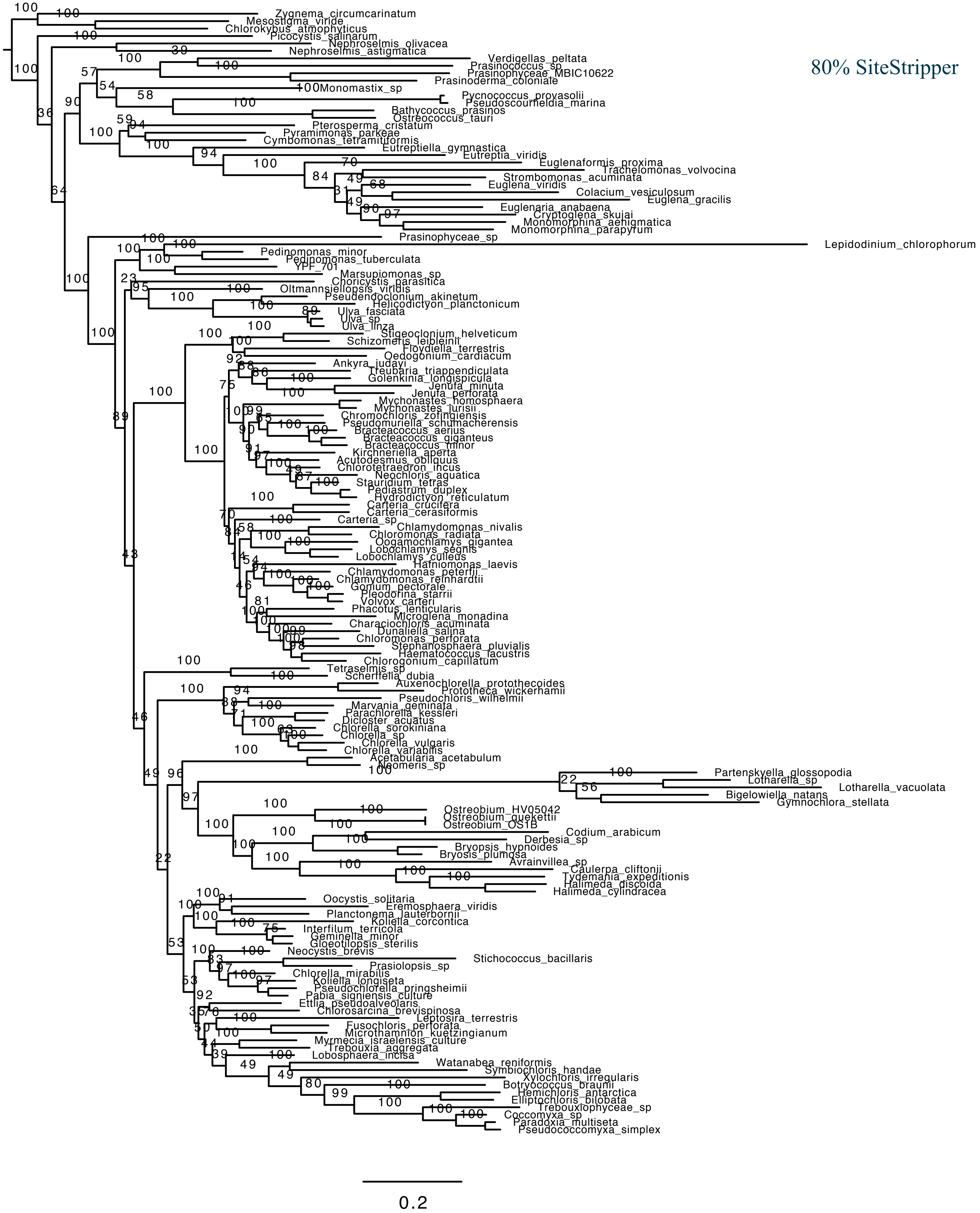
80% SiteStripper
Zygnema_circumcarinatum
Mesostigma_viride
Chlorokybus_atmophyticus
Picocystis_salinarum
Nephroselmis_olivacea
Nephroselmis_astigmatica
Verdigellas_peltata
Prasinococcus_sp
Prasinophyceae_MBIC10622
Prasinoderma_coloniale
Monomastix_sp
Pycnococcus_provasolii
Pseudoscourfieldia_marina
Bathycoccus_prasinos
Ostreococcus_tauri
Pterosperma_cristatum
Pyramimonas_parkeae
Cymbomonas_tetramitiformis
Eutreptiella_gymnastica
Eutreptia_viridis
Euglenaformis_proxima
Trachelomonas_volvocina
Strombomonas_acuminata
Euglena_viridis
Colacium_vesiculosum
Euglena_gracilis
Euglenaria_anabaena
Cryptoglena_skujai
Monomorphina_aenigmatica
Monomorphina_parapyrum
Prasinophyceae_sp
Lepidodinium_chlorophorum
Pedinomonas_minor
Pedinomonas_tuberculata
YPF_701
Marsupiomonas_sp
Choricystis_parasitica
Oltmannsiellopsis_viridis
Pseudendoclonium_akinetum
Helicodictyon_planctonicum
Ulva_fasciata
Ulva_sp
Ulva_linza
Stigeoclonium_helveticum
Schizomeris_leibleinii
Floydiella_terrestris
Oedogonium_cardiacum
Ankyra_judayi
Treubaria_triappendiculata
Golenkinia_longispicula
Jenufa_minuta
Jenufa_perforata
Mychonastes_homosphaera
Mychonastes_jurisii
Chromochloris_zofingiensis
Pseudomuriella_schumacherensis
Bracteacoccus_aerius
Bracteacoccus_giganteus
Bracteacoccus_minor
Kirchneriella_aperta
Acutodesmus_obliquus
Chlorotetraedron_incus
Neochloris_aquatica
Stauridium_tetras
Pediastrum_duplex
Hydrodictyon_reticulatum
Carteria_crucifera
Carteria_cerasiformis
Carteria_sp
Chlamydomonas_nivalis
Chloromonas_radiata
Oogamochlamys_gigantea
Lobochlamys_segnis
Lobochlamys_culleus
Hafniomonas_laevis
Chlamydomonas_peterfii
Chlamydomonas_reinhardtii
Gonium_pectorale
Pleodorina_starrii
Volvox_carteri
Phacotus_lenticularis
Microglena_monadina
Characiochloris_acuminata
Dunaliella_salina
Chloromonas_perforata
Stephanosphaera_pluvialis
Haematococcus_lacustris
Chlorogonium_capillatum
Tetraselmis_sp
Scherffelia_dubia
Auxenochlorella_protothecoides
Prototheca_wickerhamii
Pseudochloris_wilhelmii
Marvania_geminata
Parachlorella_kessleri
Dicloster_acuatus
Chlorella_sorokiniana
Chlorella_sp
Chlorella_vulgaris
Chlorella_variabilis
Acetabularia_acetabulum
Neomeris_sp
Partenskyella_glossopodia
Lotharella_sp
Lotharella_vacuolata
Bigelowiella_natans
Gymnochlora_stellata
Ostreobium_HV05042
Ostreobium_quekettii
Ostreobium_OS1B
Codium_arabicum
Derbesia_sp
Bryopsis_hypnoides
Bryosis_plumosa
Avrainvillea_sp
Caulerpa_cliftonii
Tydemania_expeditionis
Halimeda_discoida
Halimeda_cylindracea
Oocystis_solitaria
Eremosphaera_viridis
Planctonema_lauterbornii
Koliella_corcontica
Interfilum_terricola
Geminella_minor
Gloeotilopsis_sterilis
Neocystis_brevis
Stichococcus_bacillaris
Prasiolopsis_sp
Chlorella_mirabilis
Koliella_longiseta
Pseudochlorella_pringsheimii
Pabia_signiensis_culture
Ettlia_pseudoalveolaris
Chlorosarcina_brevispinosa
Leptosira_terrestris
Fusochloris_perforata
Microthamnion_kuetzingianum
Myrmecia_israelensis_culture
Trebouxia_aggregata
Lobosphaera_incisa
Watanabea_reniformis
Symbiochloris_handae
Xylochloris_irregularis
Botryococcus_braunii
Hemichloris_antarctica
Elliptochloris_bilobata
Trebouxiophyceae_sp
Coccomyxa_sp
Paradoxia_multiseta
Pseudococcomyxa_simplex
0.2

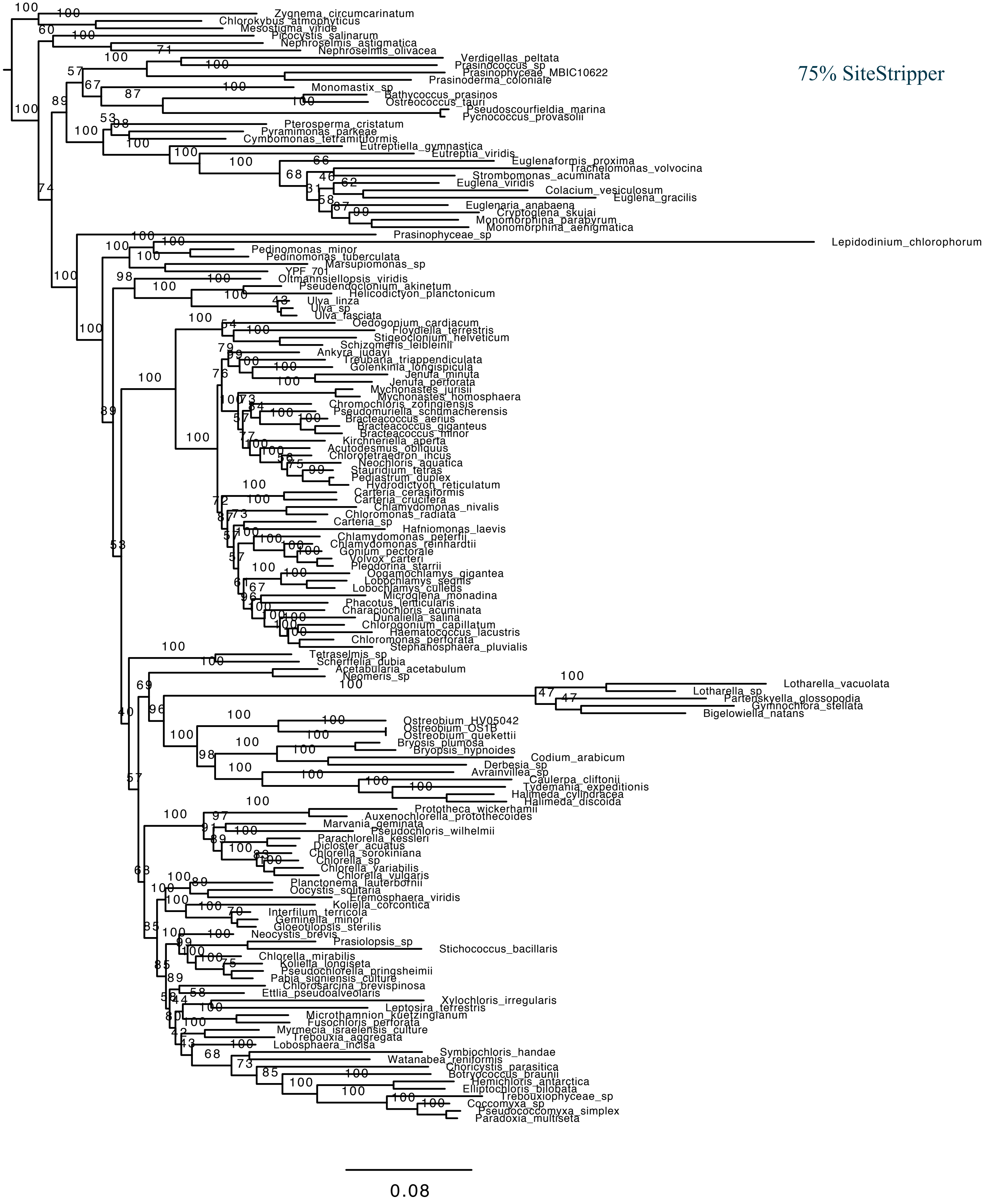
75% SiteStripper
Zygnema_circumcarinatum
Chlorokybus_atmophyticus
Mesostigma_viride
Picocystis_salinarum
Nephroselmis_astigmatica
Nephroselmis_olivacea
Verdigellas_peltata
Prasinococcus_sp
Prasinophyceae_MBIC10622
Prasinoderma_coloniale
Monomastix_sp
Bathycoccus_prasinos
Ostreococcus_tauri
Pseudoscourfieldia_marina
Pycnococcus_provasolii
Pterosperma_cristatum
Pyramimonas_parkeae
Cymbomonas_tetramitiformis
Eutreptiella_gymnastica
Eutreptia_viridis
Euglenaformis_proxima
Trachelomonas_volvocina
Strombomonas_acuminata
Euglena_viridis
Colacium_vesiculosum
Euglena_gracilis
Euglenaria_anabaena
Cryptoglena_skujai
Monomorphina_parapyrum
Monomorphina_aenigmatica
Prasinophyceae_sp
Lepidodinium_chlorophorum
Pedinomonas_minor
Pedinomonas_tuberculata
Marsupiomonas_sp
YPF_701
Oltmannsiellopsis_viridis
Pseudendoclonium_akinetum
Helicodictyon_planctonicum
Ulva_linza
Ulva_sp
Ulva_fasciata
Oedogonium_cardiacum
Floydiella_terrestris
Stigeoclonium_helveticum
Schizomeris_leibleinii
Ankyra_judayi
Treubaria_triappendiculata
Golenkinia_longispicula
Jenufa_minuta
Jenufa_perforata
Mychonastes_jurisii
Mychonastes_homosphaera
Chromochloris_zofingiensis
Pseudomuriella_schumacherensis
Bracteacoccus_aerius
Bracteacoccus_giganteus
Bracteacoccus_minor
Kirchneriella_aperta
Acutodesmus_obliquus
Chlorotetraedron_incus
Neochloris_aquatica
Stauridium_tetras
Pediastrum_duplex
Hydrodictyon_reticulatum
Carteria_cerasiformis
Carteria_crucifera
Chlamydomonas_nivalis
Chloromonas_radiata
Carteria_sp
Hafniomonas_laevis
Chlamydomonas_peterfii
Chlamydomonas_reinhardtii
Gonium_pectorale
Volvox_carteri
Pleodorina_starrii
Oogamochlamys_gigantea
Lobochlamys_segnis
Lobochlamys_culleus
Microglena_monadina
Phacotus_lenticularis
Characiochloris_acuminata
Dunaliella_salina
Chlorogonium_capillatum
Haematococcus_lacustris
Chloromonas_perforata
Stephanosphaera_pluvialis
Tetraselmis_sp
Scherffelia_dubia
Acetabularia_acetabulum
Neomeris_sp
Lotharella_vacuolata
Lotharella_sp
Partenskyella_glossopodia
Gymnochlora_stellata
Bigelowiella_natans
Ostreobium_HV05042
Ostreobium_OS1B
Ostreobium_quekettii
Bryopsis_plumosa
Bryopsis_hypnoides
Codium_arabicum
Derbesia_sp
Avrainvillea_sp
Caulerpa_cliftonii
Tydemania_expeditionis
Halimeda_cylindracea
Halimeda_discoida
Prototheca_wickerhamii
Auxenochlorella_protothecoides
Marvania_geminata
Pseudochloris_wilhelmii
Parachlorella_kessleri
Dicloster_acuatus
Chlorella_sorokiniana
Chlorella_sp
Chlorella_variabilis
Chlorella_vulgaris
Planctonema_lauterbornii
Oocystis_solitaria
Eremosphaera_viridis
Koliella_corcontica
Interfilum_terricola
Geminella_minor
Gloeotilopsis_sterilis
Neocystis_brevis
Prasiolopsis_sp
Stichococcus_bacillaris
Chlorella_mirabilis
Koliella_longiseta
Pseudochlorella_pringsheimii
Pabia_signiensis_culture
Chlorosarcina_brevispinosa
Ettlia_pseudoalveolaris
Xylochloris_irregularis
Leptosira_terrestris
Microthamnion_kuetzingianum
Fusochloris_perforata
Myrmecia_israelensis_culture
Trebouxia_aggregata
Lobosphaera_incisa
Symbiochloris_handae
Watanabea_reniformis
Choricystis_parasitica
Botryococcus_braunii
Hemichloris_antarctica
Elliptochloris_bilobata
Trebouxiophyceae_sp
Coccomyxa_sp
Pseudococcomyxa_simplex
Paradoxia_multiseta
0.08

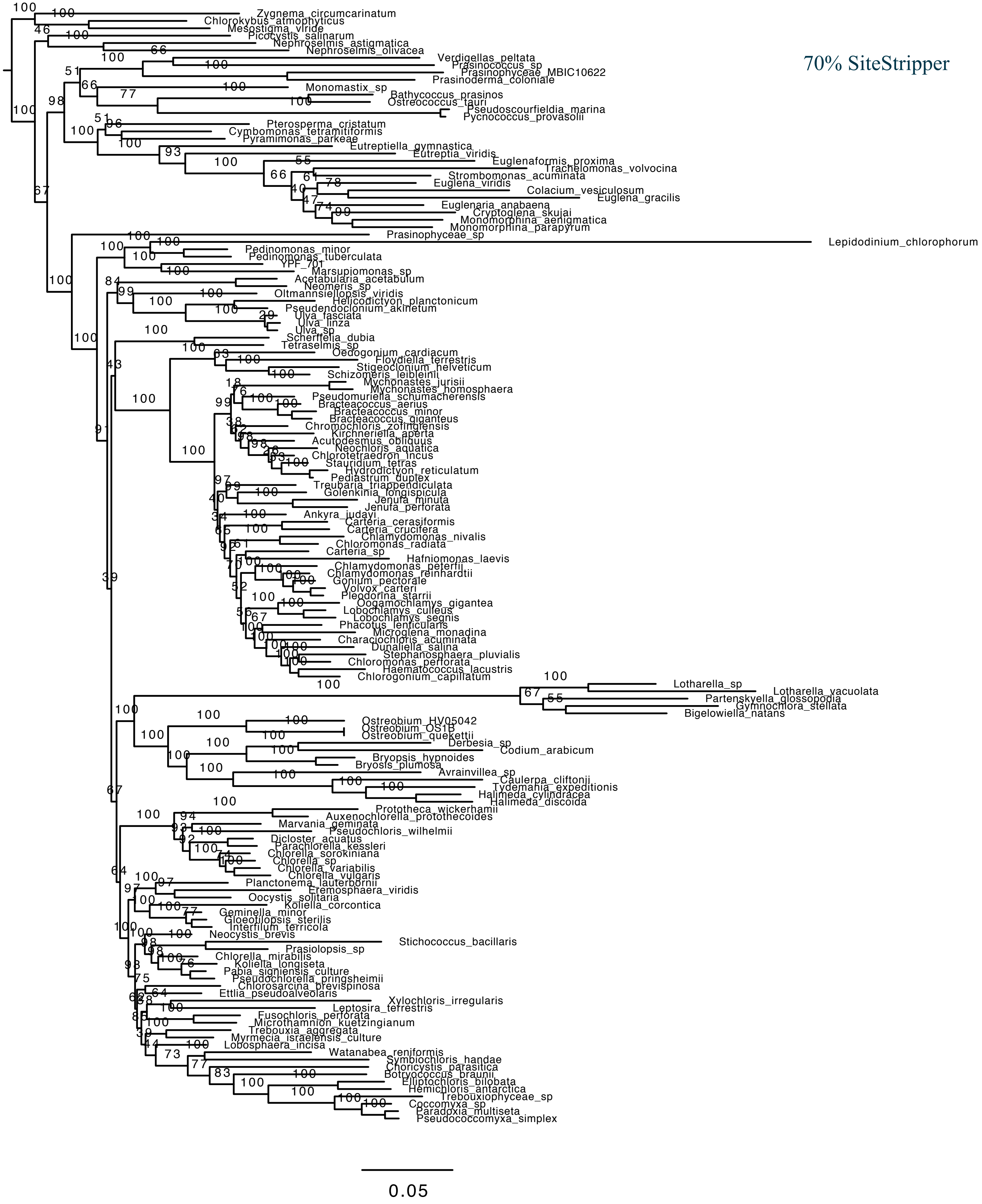
70% SiteStripper
Zygnema_circumcarinatum
Chlorokybus_atmophyticus
Mesostigma_viride
Picocystis_salinarum
Nephroselmis_astigmatica
Nephroselmis_olivacea
Verdigellas_peltata
Prasinococcus_sp
Prasinophyceae_MBIC10622
Prasinoderma_coloniale
Monomastix_sp
Bathycoccus_prasinos
Ostreococcus_tauri
Pseudoscourfieldia_marina
Pycnococcus_provasolii
Pterosperma_cristatum
Cymbomonas_tetramitiformis
Pyramimonas_parkeae
Eutreptiella_gymnastica
Eutreptia_viridis
Euglenaformis_proxima
Trachelomonas_volvocina
Strombomonas_acuminata
Euglena_viridis
Colacium_vesiculosum
Euglena_gracilis
Euglenaria_anabaena
Cryptoglena_skujai
Monomorphina_aenigmatica
Monomorphina_parapyrum
Prasinophyceae_sp
Lepidodinium_chlorophorum
Pedinomonas_minor
Pedinomonas_tuberculata
YPF_701
Marsupiomonas_sp
Acetabularia_acetabulum
Neomeris_sp
Oltmannsiellopsis_viridis
Helicodictyon_planctonicum
Pseudendoclonium_akinetum
Ulva_fasciata
Ulva_linza
Ulva_sp
Scherffelia_dubia
Tetraselmis_sp
Oedogonium_cardiacum
Floydiella_terrestris
Stigeoclonium_helveticum
Schizomeris_leibleinii
Mychonastes_jurisii
Mychonastes_homosphaera
Pseudomuriella_schumacherensis
Bracteacoccus_aerius
Bracteacoccus_minor
Bracteacoccus_giganteus
Chromochloris_zofingiensis
Kirchneriella_aperta
Acutodesmus_obliquus
Neochloris_aquatica
Chlorotetraedron_incus
Stauridium_tetras
Hydrodictyon_reticulatum
Pediastrum_duplex
Treubaria_triappendiculata
Golenkinia_longispicula
Jenufa_minuta
Jenufa_perforata
Ankyra_judayi
Carteria_cerasiformis
Carteria_crucifera
Chlamydomonas_nivalis
Chloromonas_radiata
Carteria_sp
Hafniomonas_laevis
Chlamydomonas_peterfii
Chlamydomonas_reinhardtii
Gonium_pectorale
Volvox_carteri
Pleodorina_starrii
Oogamochlamys_gigantea
Lobochlamys_culleus
Lobochlamys_segnis
Phacotus_lenticularis
Microglena_monadina
Characiochloris_acuminata
Dunaliella_salina
Stephanosphaera_pluvialis
Chloromonas_perforata
Haematococcus_lacustris
Chlorogonium_capillatum
Lotharella_sp
Lotharella_vacuolata
Partenskyella_glossopodia
Gymnochlora_stellata
Bigelowiella_natans
Ostreobium_HV05042
Ostreobium_OS1B
Ostreobium_quekettii
Derbesia_sp
Codium_arabicum
Bryopsis_hypnoides
Bryosis_plumosa
Avrainvillea_sp
Caulerpa_cliftonii
Tydemania_expeditionis
Halimeda_cylindracea
Halimeda_discoida
Prototheca_wickerhamii
Auxenochlorella_protothecoides
Marvania_geminata
Pseudochloris_wilhelmii
Dicloster_acuatus
Parachlorella_kessleri
Chlorella_sorokiniana
Chlorella_sp
Chlorella_variabilis
Chlorella_vulgaris
Planctonema_lauterbornii
Eremosphaera_viridis
Oocystis_solitaria
Koliella_corcontica
Geminella_minor
Gloeotilopsis_sterilis
Interfilum_terricola
Neocystis_brevis
Stichococcus_bacillaris
Prasiolopsis_sp
Chlorella_mirabilis
Koliella_longiseta
Pabia_signiensis_culture
Pseudochlorella_pringsheimii
Chlorosarcina_brevispinosa
Ettlia_pseudoalveolaris
Xylochloris_irregularis
Leptosira_terrestris
Fusochloris_perforata
Microthamnion_kuetzingianum
Trebouxia_aggregata
Myrmecia_israelensis_culture
Lobosphaera_incisa
Watanabea_reniformis
Symbiochloris_handae
Choricystis_parasitica
Botryococcus_braunii
Elliptochloris_bilobata
Hemichloris_antarctica
Trebouxiophyceae_sp
Coccomyxa_sp
Paradoxia_multiseta
Pseudococcomyxa_simplex
0.05

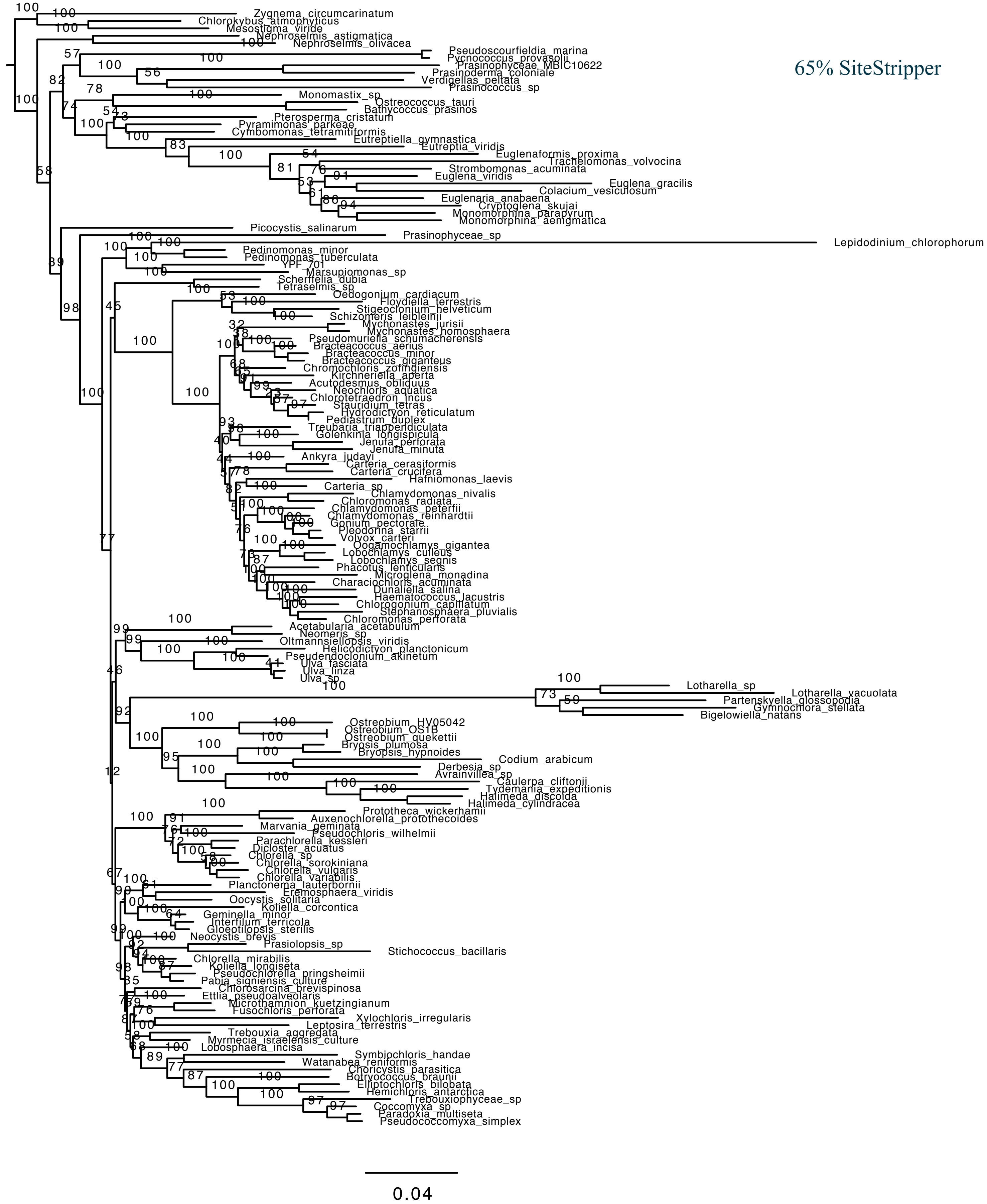

65% SiteStripper
Zygnema_circumcarinatum
Chlorokybus_atmophyticus
Mesostigma_viride
Nephroselmis_astigmatica
Nephroselmis_olivacea
Pseudoscourfieldia_marina
Pycnococcus_provasolii
Prasinophyceae_MBIC10622
Prasinoderma_coloniale
Verdigellas_peltata
Prasinococcus_sp
Monomastix_sp
Ostreococcus_tauri
Bathycoccus_prasinos
Pterosperma_cristatum
Pyramimonas_parkeae
Cymbomonas_tetramitiformis
Eutreptiella_gymnastica
Eutreptia_viridis
Euglenaformis_proxima
Trachelomonas_volvocina
Strombomonas_acuminata
Euglena_viridis
Euglena_gracilis
Colacium_vesiculosum
Euglenaria_anabaena
Cryptoglena_skujai
Monomorphina_parapyrum
Monomorphina_aenigmatica
Picocystis_salinarum
Prasinophyceae_sp
Lepidodinium_chlorophorum
Pedinomonas_minor
Pedinomonas_tuberculata
YPF_701
Marsupiomonas_sp
Scherffelia_dubia
Tetraselmis_sp
Oedogonium_cardiacum
Floydiella_terrestris
Stigeoclonium_helveticum
Schizomeris_leibleinii
Mychonastes_jurisii
Mychonastes_homosphaera
Pseudomuriella_schumacherensis
Bracteacoccus_aerius
Bracteacoccus_minor
Bracteacoccus_giganteus
Chromochloris_zofingiensis
Kirchneriella_aperta
Acutodesmus_obliquus
Neochloris_aquatica
Chlorotetraedron_incus
Stauridium_tetras
Hydrodictyon_reticulatum
Pediastrum_duplex
Treubaria_triappendiculata
Golenkinia_longispicula
Jenufa_perforata
Jenufa_minuta
Ankyra_judayi
Carteria_cerasiformis
Carteria_crucifera
Hafniomonas_laevis
Carteria_sp
Chlamydomonas_nivalis
Chloromonas_radiata
Chlamydomonas_peterfii
Chlamydomonas_reinhardtii
Gonium_pectorale
Pleodorina_starrii
Volvox_carteri
Oogamochlamys_gigantea
Lobochlamys_culleus
Lobochlamys_segnis
Phacotus_lenticularis
Microglena_monadina
Characiochloris_acuminata
Dunaliella_salina
Haematococcus_lacustris
Chlorogonium_capillatum
Stephanosphaera_pluvialis
Chloromonas_perforata
Acetabularia_acetabulum
Neomeris_sp
Oltmannsiellopsis_viridis
Helicodictyon_planctonicum
Pseudendoclonium_akinetum
Ulva_fasciata
Ulva_linza
Ulva_sp
Lotharella_sp
Lotharella_vacuolata
Partenskyella_glossopodia
Gymnochlora_stellata
Bigelowiella_natans
Ostreobium_HV05042
Ostreobium_OS1B
Ostreobium_quekettii
Bryopsis_plumosa
Bryopsis_hypnoides
Codium_arabicum
Derbesia_sp
Avrainvillea_sp
Caulerpa_cliftonii
Tydemania_expeditionis
Halimeda_discoida
Halimeda_cylindracea
Prototheca_wickerhamii
Auxenochlorella_protothecoides
Marvania_geminata
Pseudochloris_wilhelmii
Parachlorella_kessleri
Dicloster_acuatus
Chlorella_sp
Chlorella_sorokiniana
Chlorella_vulgaris
Chlorella_variabilis
Planctonema_lauterbornii
Eremosphaera_viridis
Oocystis_solitaria
Koliella_corcontica
Geminella_minor
Interfilum_terricola
Gloeotilopsis_sterilis
Neocystis_brevis
Prasiolopsis_sp
Stichococcus_bacillaris
Chlorella_mirabilis
Koliella_longiseta
Pseudochlorella_pringsheimii
Pabia_signiensis_culture
Chlorosarcina_brevispinosa
Ettlia_pseudoalveolaris
Microthamnion_kuetzingianum
Fusochloris_perforata
Xylochloris_irregularis
Leptosira_terrestris
Trebouxia_aggregata
Myrmecia_israelensis_culture
Lobosphaera_incisa
Symbiochloris_handae
Watanabea_reniformis
Choricystis_parasitica
Botryococcus_braunii
Elliptochloris_bilobata
Hemichloris_antarctica
Trebouxiophyceae_sp
Coccomyxa_sp
Paradoxia_multiseta
Pseudococcomyxa_simplex
0.04

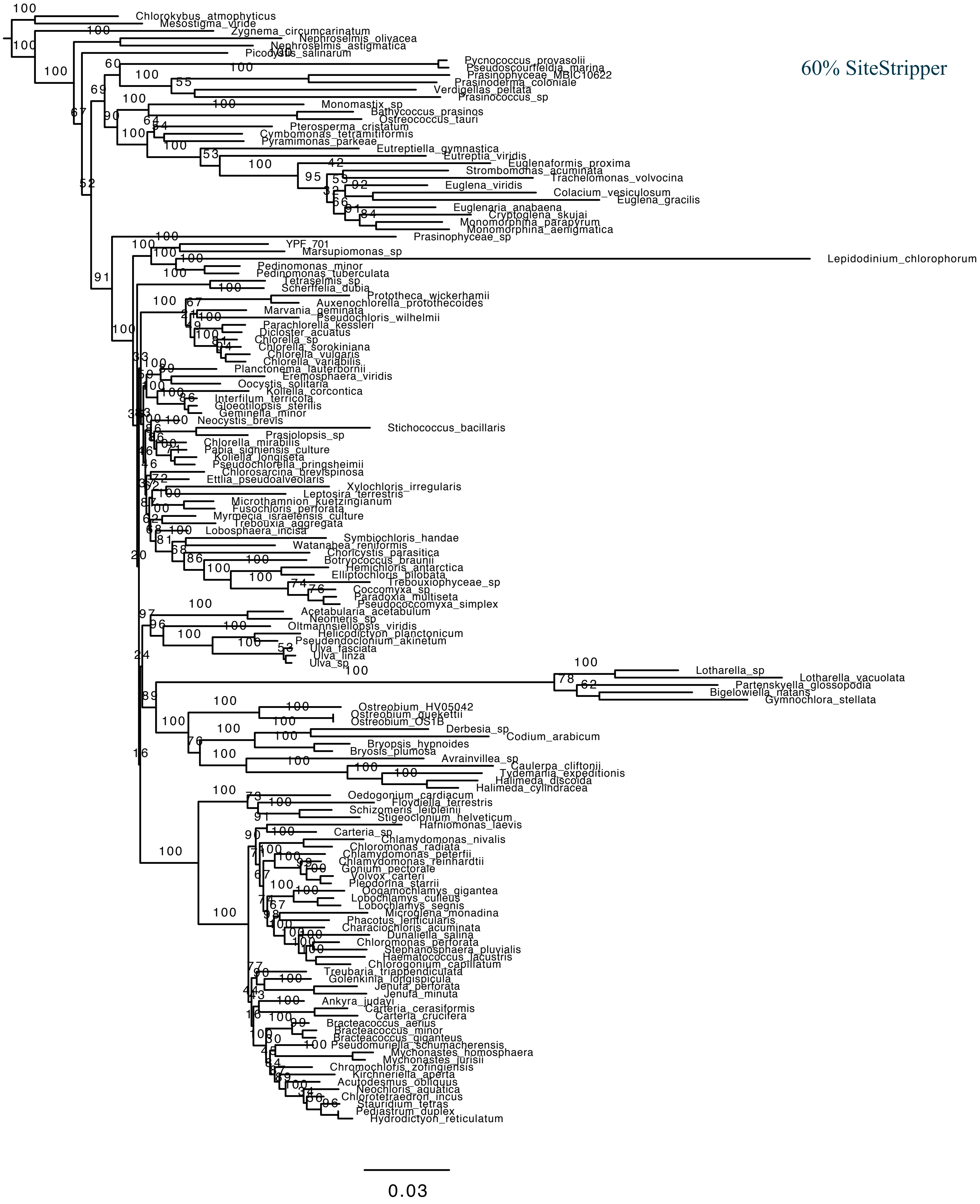
60% SiteStripper
Chlorokybus_atmophyticus
Mesostigma_viride
Zygnema_circumcarinatum
Nephroselmis_olivacea
Nephroselmis_astigmatica
Picocystis_salinarum
Pycnococcus_provasolii
Pseudoscourfieldia_marina
Prasinophyceae_MBIC10622
Prasinoderma_coloniale
Verdigellas_peltata
Prasinococcus_sp
Monomastix_sp
Bathycoccus_prasinos
Ostreococcus_tauri
Pterosperma_cristatum
Cymbomonas_tetramitiformis
Pyramimonas_parkeae
Eutreptiella_gymnastica
Eutreptia_viridis
Euglenaformis_proxima
Strombomonas_acuminata
Trachelomonas_volvocina
Euglena_viridis
Colacium_vesiculosum
Euglena_gracilis
Euglenaria_anabaena
Cryptoglena_skujai
Monomorphina_parapyrum
Monomorphina_aenigmatica
Prasinophyceae_sp
YPF_701
Marsupiomonas_sp
Lepidodinium_chlorophorum
Pedinomonas_minor
Pedinomonas_tuberculata
Tetraselmis_sp
Scherffelia_dubia
Prototheca_wickerhamii
Auxenochlorella_protothecoides
Marvania_geminata
Pseudochloris_wilhelmii
Parachlorella_kessleri
Dicloster_acuatus
Chlorella_sp
Chlorella_sorokiniana
Chlorella_vulgaris
Chlorella_variabilis
Planctonema_lauterbornii
Eremosphaera_viridis
Oocystis_solitaria
Koliella_corcontica
Interfilum_terricola
Gloeotilopsis_sterilis
Geminella_minor
Neocystis_brevis
Stichococcus_bacillaris
Prasiolopsis_sp
Chlorella_mirabilis
Pabia_signiensis_culture
Koliella_longiseta
Pseudochlorella_pringsheimii
Chlorosarcina_brevispinosa
Ettlia_pseudoalveolaris
Xylochloris_irregularis
Leptosira_terrestris
Microthamnion_kuetzingianum
Fusochloris_perforata
Myrmecia_israelensis_culture
Trebouxia_aggregata
Lobosphaera_incisa
Symbiochloris_handae
Watanabea_reniformis
Choricystis_parasitica
Botryococcus_braunii
Hemichloris_antarctica
Elliptochloris_bilobata
Trebouxiophyceae_sp
Coccomyxa_sp
Paradoxia_multiseta
Pseudococcomyxa_simplex
Acetabularia_acetabulum
Neomeris_sp
Oltmannsiellopsis_viridis
Helicodictyon_planctonicum
Pseudendoclonium_akinetum
Ulva_fasciata
Ulva_linza
Ulva_sp
Lotharella_sp
Lotharella_vacuolata
Partenskyella_glossopodia
Bigelowiella_natans
Gymnochlora_stellata
Ostreobium_HV05042
Ostreobium_quekettii
Ostreobium_OS1B
Derbesia_sp
Codium_arabicum
Bryopsis_hypnoides
Bryosis_plumosa
Avrainvillea_sp
Caulerpa_cliftonii
Tydemania_expeditionis
Halimeda_discoida
Halimeda_cylindracea
Oedogonium_cardiacum
Floydiella_terrestris
Schizomeris_leibleinii
Stigeoclonium_helveticum
Hafniomonas_laevis
Carteria_sp
Chlamydomonas_nivalis
Chloromonas_radiata
Chlamydomonas_peterfii
Chlamydomonas_reinhardtii
Gonium_pectorale
Volvox_carteri
Pleodorina_starrii
Oogamochlamys_gigantea
Lobochlamys_culleus
Lobochlamys_segnis
Microglena_monadina
Phacotus_lenticularis
Characiochloris_acuminata
Dunaliella_salina
Chloromonas_perforata
Stephanosphaera_pluvialis
Haematococcus_lacustris
Chlorogonium_capillatum
Treubaria_triappendiculata
Golenkinia_longispicula
Jenufa_perforata
Jenufa_minuta
Ankyra_judayi
Carteria_cerasiformis
Carteria_crucifera
Bracteacoccus_aerius
Bracteacoccus_minor
Bracteacoccus_giganteus
Pseudomuriella_schumacherensis
Mychonastes_homosphaera
Mychonastes_jurisii
Chromochloris_zofingiensis
Kirchneriella_aperta
Acutodesmus_obliquus
Neochloris_aquatica
Chlorotetraedron_incus
Stauridium_tetras
Pediastrum_duplex
Hydrodictyon_reticulatum
0.03

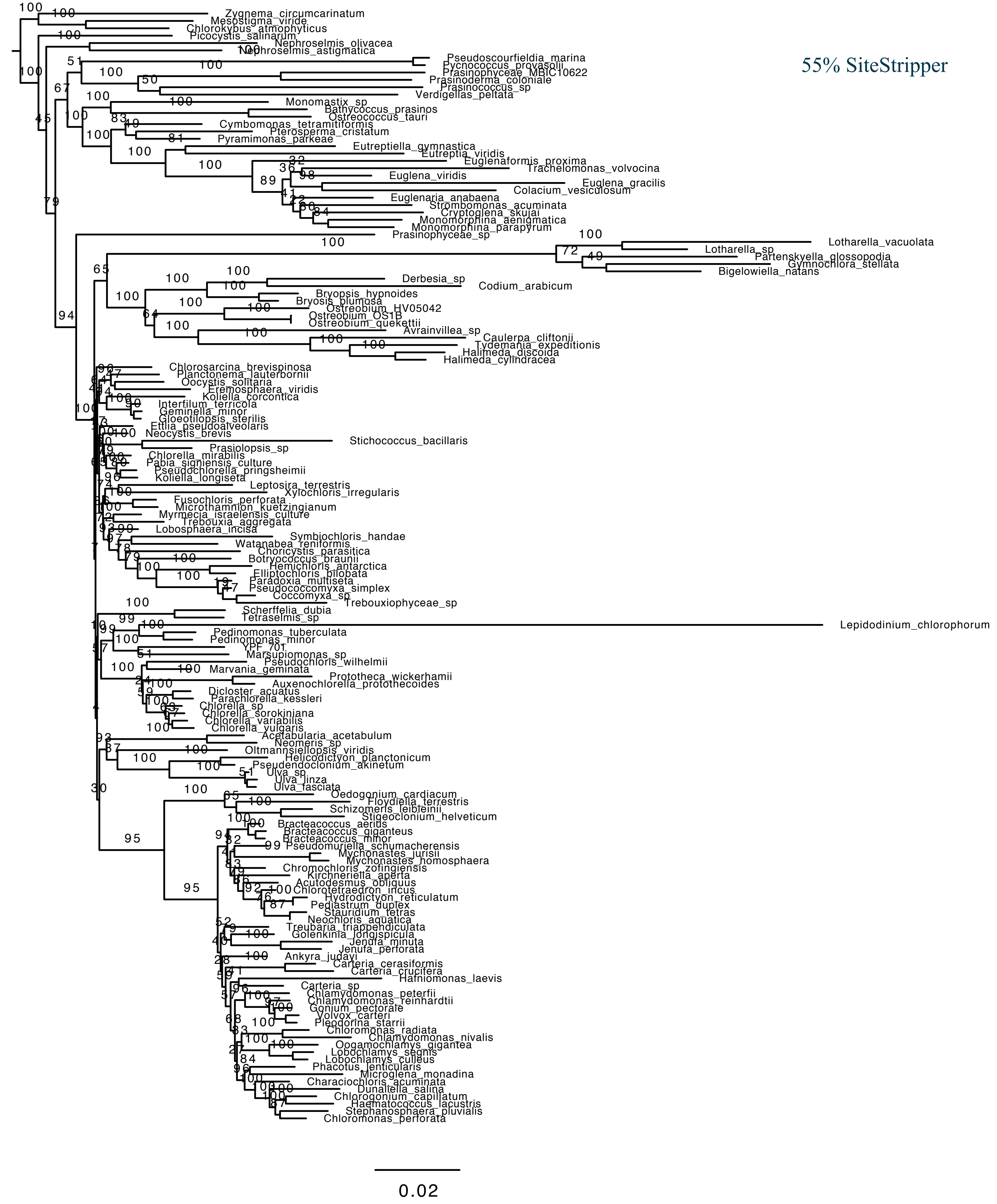
55% SiteStripper
Zygnema_circumcarinatum
Mesostigma_viride
Chlorokybus_atmophyticus
Picocystis_salinarum
Nephroselmis_olivacea
Nephroselmis_astigmatica
Pseudoscourfieldia_marina
Pycnococcus_provasolii
Prasinophyceae_MBIC10622
Prasinoderma_coloniale
Prasinococcus_sp
Verdigellas_peltata
Monomastix_sp
Bathycoccus_prasinos
Ostreococcus_tauri
Cymbomonas_tetramitiformis
Pterosperma_cristatum
Pyramimonas_parkeae
Eutreptiella_gymnastica
Eutreptia_viridis
Euglenaformis_proxima
Trachelomonas_volvocina
Euglena_viridis
Euglena_gracilis
Colacium_vesiculosum
Euglenaria_anabaena
Strombomonas_acuminata
Cryptoglena_skujai
Monomorphina_aenigmatica
Monomorphina_parapyrum
Prasinophyceae_sp
Lotharella_vacuolata
Lotharella_sp
Partenskyella_glossopodia
Gymnochlora_stellata
Bigelowiella_natans
Derbesia_sp
Codium_arabicum
Bryopsis_hypnoides
Bryosis_plumosa
Ostreobium_HV05042
Ostreobium_OS1B
Ostreobium_quekettii
Avrainvillea_sp
Caulerpa_cliftonii
Tydemania_expeditionis
Halimeda_discoida
Halimeda_cylindracea
Chlorosarcina_brevispinosa
Planctonema_lauterbornii
Oocystis_solitaria
Eremosphaera_viridis
Koliella_corcontica
Interfilum_terricola
Geminella_minor
Gloeotilopsis_sterilis
Ettlia_pseudoalveolaris
Neocystis_brevis
Stichococcus_bacillaris
Prasiolopsis_sp
Chlorella_mirabilis
Pabia_signiensis_culture
Pseudochlorella_pringsheimii
Koliella_longiseta
Leptosira_terrestris
Xylochloris_irregularis
Fusochloris_perforata
Microthamnion_kuetzingianum
Myrmecia_israelensis_culture
Trebouxia_aggregata
Lobosphaera_incisa
Symbiochloris_handae
Watanabea_reniformis
Choricystis_parasitica
Botryococcus_braunii
Hemichloris_antarctica
Elliptochloris_bilobata
Paradoxia_multiseta
Pseudococcomyxa_simplex
Coccomyxa_sp
Trebouxiophyceae_sp
Scherffelia_dubia
Tetraselmis_sp
Lepidodinium_chlorophorum
Pedinomonas_tuberculata
Pedinomonas_minor
YPF_701
Marsupiomonas_sp
Pseudochloris_wilhelmii
Marvania_geminata
Prototheca_wickerhamii
Auxenochlorella_protothecoides
Dicloster_acuatus
Parachlorella_kessleri
Chlorella_sp
Chlorella_sorokiniana
Chlorella_variabilis
Chlorella_vulgaris
Acetabularia_acetabulum
Neomeris_sp
Oltmannsiellopsis_viridis
Helicodictyon_planctonicum
Pseudendoclonium_akinetum
Ulva_sp
Ulva_linza
Ulva_fasciata
Oedogonium_cardiacum
Floydiella_terrestris
Schizomeris_leibleinii
Stigeoclonium_helveticum
Bracteacoccus_aerius
Bracteacoccus_giganteus
Bracteacoccus_minor
Pseudomuriella_schumacherensis
Mychonastes_jurisii
Mychonastes_homosphaera
Chromochloris_zofingiensis
Kirchneriella_aperta
Acutodesmus_obliquus
Chlorotetraedron_incus
Hydrodictyon_reticulatum
Pediastrum_duplex
Stauridium_tetras
Neochloris_aquatica
Treubaria_triappendiculata
Golenkinia_longispicula
Jenufa_minuta
Jenufa_perforata
Ankyra_judayi
Carteria_cerasiformis
Carteria_crucifera
Hafniomonas_laevis
Carteria_sp
Chlamydomonas_peterfii
Chlamydomonas_reinhardtii
Gonium_pectorale
Volvox_carteri
Pleodorina_starrii
Chloromonas_radiata
Chlamydomonas_nivalis
Oogamochlamys_gigantea
Lobochlamys_segnis
Lobochlamys_culleus
Phacotus_lenticularis
Microglena_monadina
Characiochloris_acuminata
Dunaliella_salina
Chlorogonium_capillatum
Haematococcus_lacustris
Stephanosphaera_pluvialis
Chloromonas_perforata
0.02

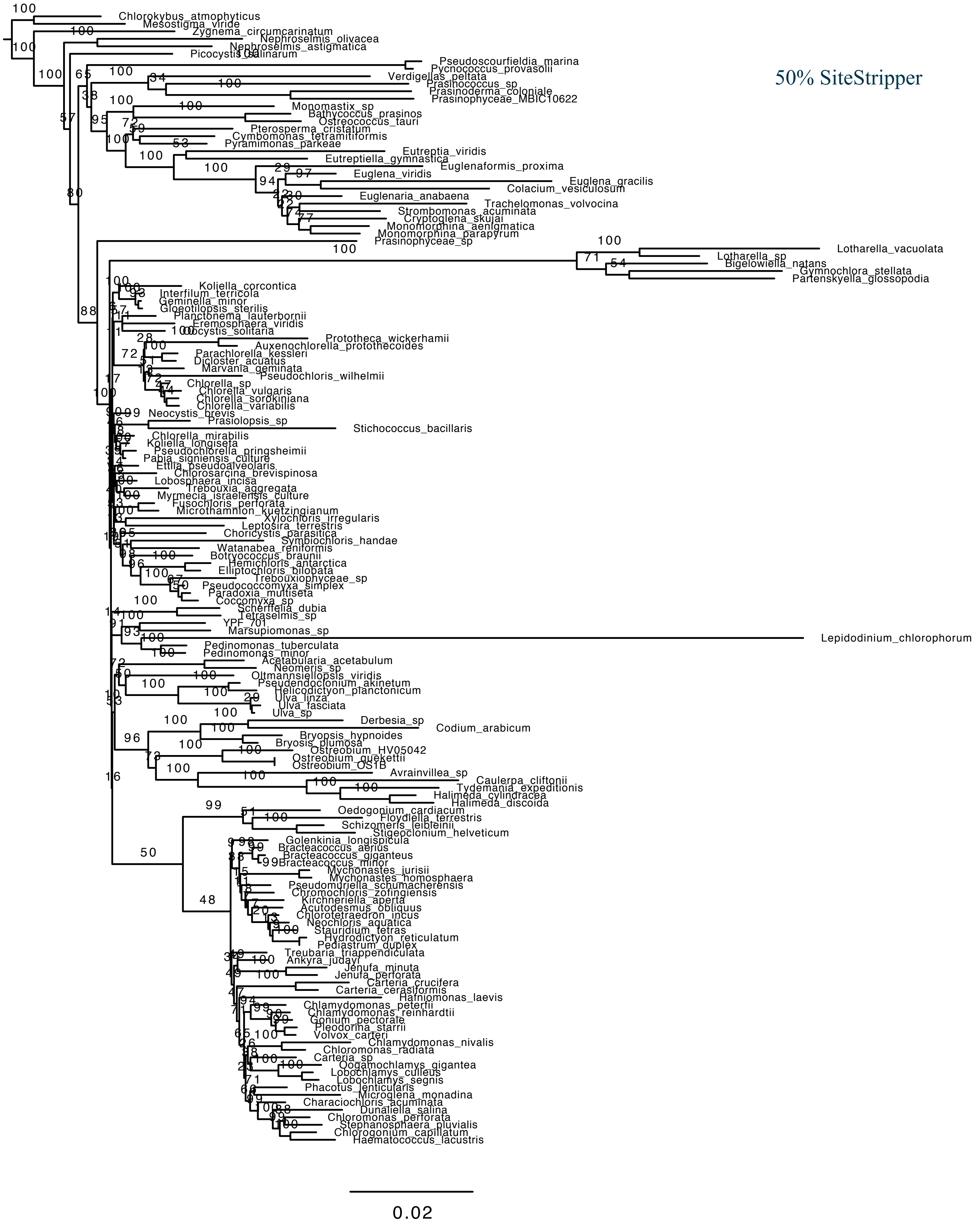
50% SiteStripper
Chlorokybus_atmophyticus
Mesostigma_viride
Zygnema_circumcarinatum
Nephroselmis_olivacea
Nephroselmis_astigmatica
Picocystis_salinarum
Pseudoscourfieldia_marina
Pycnococcus_provasolii
Verdigellas_peltata
Prasinococcus_sp
Prasinoderma_coloniale
Prasinophyceae_MBIC10622
Monomastix_sp
Bathycoccus_prasinos
Ostreococcus_tauri
Pterosperma_cristatum
Cymbomonas_tetramitiformis
Pyramimonas_parkeae
Eutreptia_viridis
Eutreptiella_gymnastica
Euglenaformis_proxima
Euglena_viridis
Euglena_gracilis
Colacium_vesiculosum
Euglenaria_anabaena
Trachelomonas_volvocina
Strombomonas_acuminata
Cryptoglena_skujai
Monomorphina_aenigmatica
Monomorphina_parapyrum
Prasinophyceae_sp
Lotharella_vacuolata
Lotharella_sp
Bigelowiella_natans
Gymnochlora_stellata
Partenskyella_glossopodia
Koliella_corcontica
Interfilum_terricola
Geminella_minor
Gloeotilopsis_sterilis
Planctonema_lauterbornii
Eremosphaera_viridis
Oocystis_solitaria
Prototheca_wickerhamii
Auxenochlorella_protothecoides
Parachlorella_kessleri
Dicloster_acuatus
Marvania_geminata
Pseudochloris_wilhelmii
Chlorella_sp
Chlorella_vulgaris
Chlorella_sorokiniana
Chlorella_variabilis
Neocystis_brevis
Prasiolopsis_sp
Stichococcus_bacillaris
Chlorella_mirabilis
Koliella_longiseta
Pseudochlorella_pringsheimii
Pabia_signiensis_culture
Ettlia_pseudoalveolaris
Chlorosarcina_brevispinosa
Lobosphaera_incisa
Trebouxia_aggregata
Myrmecia_israelensis_culture
Fusochloris_perforata
Microthamnion_kuetzingianum
Xylochloris_irregularis
Leptosira_terrestris
Choricystis_parasitica
Symbiochloris_handae
Watanabea_reniformis
Botryococcus_braunii
Hemichloris_antarctica
Elliptochloris_bilobata
Trebouxiophyceae_sp
Pseudococcomyxa_simplex
Paradoxia_multiseta
Coccomyxa_sp
Scherffelia_dubia
Tetraselmis_sp
YPF_701
Marsupiomonas_sp
Lepidodinium_chlorophorum
Pedinomonas_tuberculata
Pedinomonas_minor
Acetabularia_acetabulum
Neomeris_sp
Oltmannsiellopsis_viridis
Pseudendoclonium_akinetum
Helicodictyon_planctonicum
Ulva_linza
Ulva_fasciata
Ulva_sp
Derbesia_sp
Codium_arabicum
Bryopsis_hypnoides
Bryosis_plumosa
Ostreobium_HV05042
Ostreobium_quekettii
Ostreobium_OS1B
Avrainvillea_sp
Caulerpa_cliftonii
Tydemania_expeditionis
Halimeda_cylindracea
Halimeda_discoida
Oedogonium_cardiacum
Floydiella_terrestris
Schizomeris_leibleinii
Stigeoclonium_helveticum
Golenkinia_longispicula
Bracteacoccus_aerius
Bracteacoccus_giganteus
Bracteacoccus_minor
Mychonastes_jurisii
Mychonastes_homosphaera
Pseudomuriella_schumacherensis
Chromochloris_zofingiensis
Kirchneriella_aperta
Acutodesmus_obliquus
Chlorotetraedron_incus
Neochloris_aquatica
Stauridium_tetras
Hydrodictyon_reticulatum
Pediastrum_duplex
Treubaria_triappendiculata
Ankyra_judayi
Jenufa_minuta
Jenufa_perforata
Carteria_crucifera
Carteria_cerasiformis
Hafniomonas_laevis
Chlamydomonas_peterfii
Chlamydomonas_reinhardtii
Gonium_pectorale
Pleodorina_starrii
Volvox_carteri
Chlamydomonas_nivalis
Chloromonas_radiata
Carteria_sp
Oogamochlamys_gigantea
Lobochlamys_culleus
Lobochlamys_segnis
Phacotus_lenticularis
Microglena_monadina
Characiochloris_acuminata
Dunaliella_salina
Chloromonas_perforata
Stephanosphaera_pluvialis
Chlorogonium_capillatum
Haematococcus_lacustris
0.02

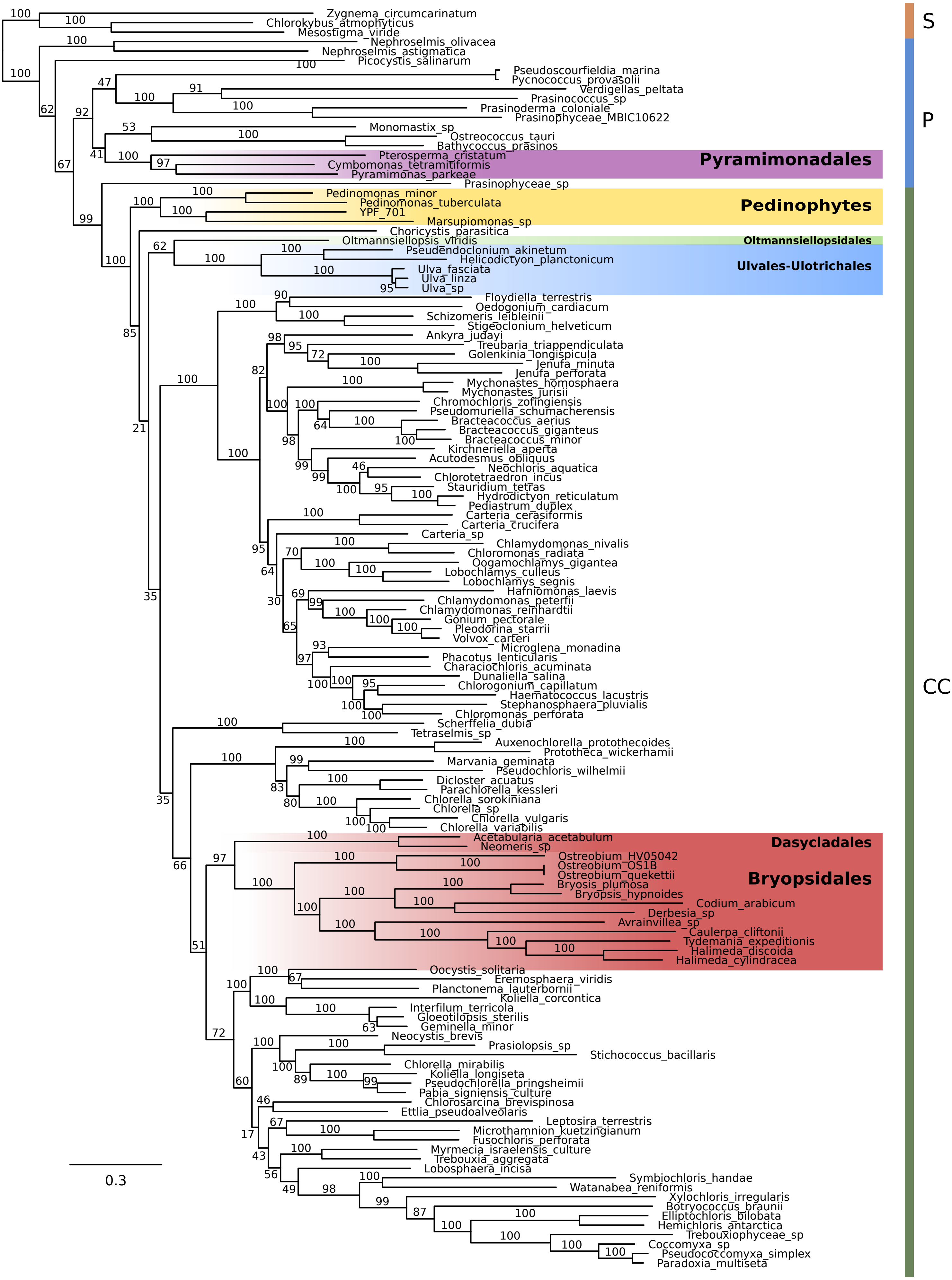

Figure S9. RAxML phylogenetic analysis (GTR model, partitioned by gene and codon position via PartitionFinder) inferred from a nucleotide alignment of 64 plastid genes from streptophytes and green algae. Coloured vertical bars to the right of the phylogeny are labelled: S, Streptophytes; P, prasinophytes; CC, core Chlorophyta. Branch lengths are proportional to the number of substitution per site.

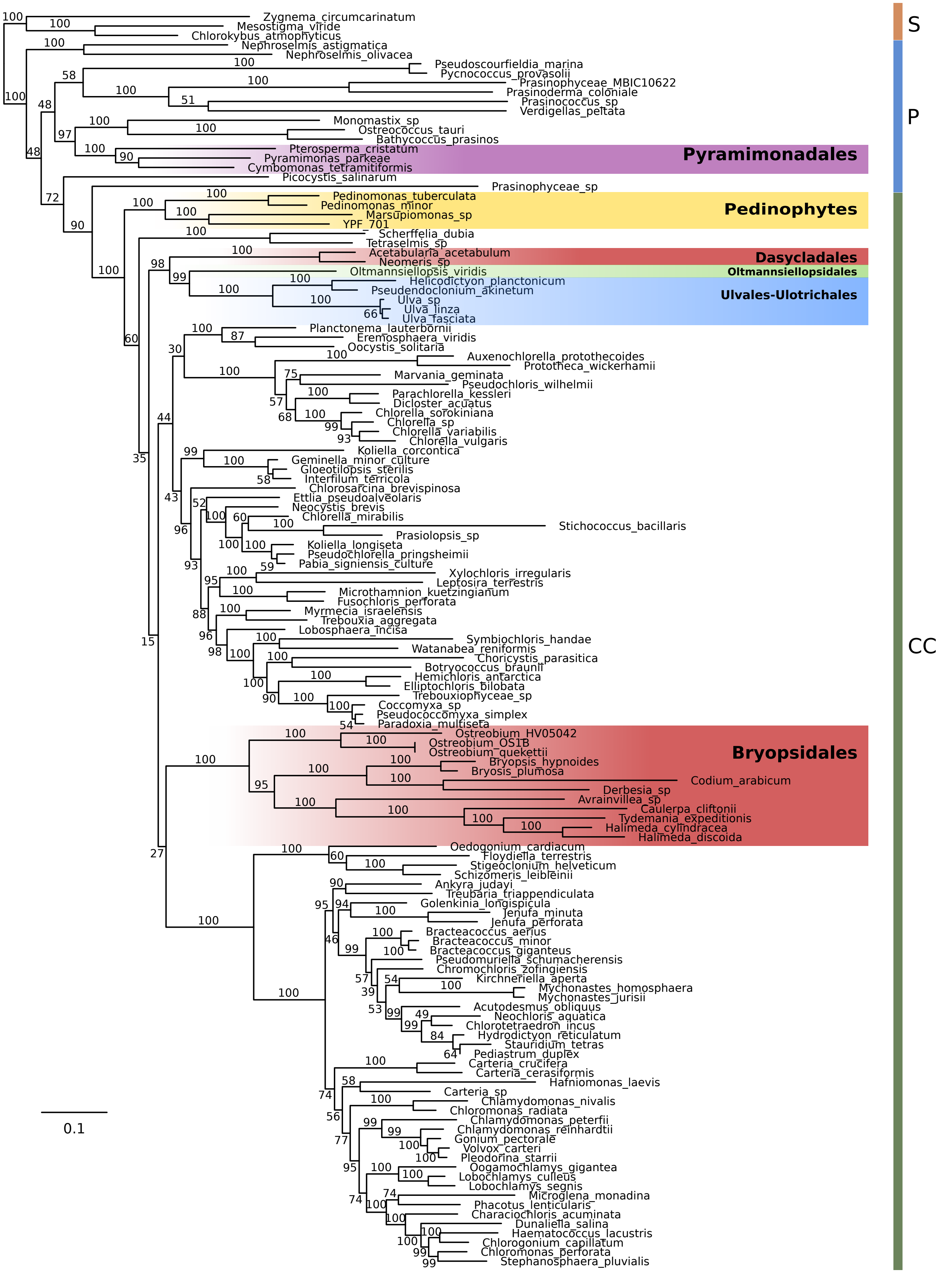

Figure S10. RAxML phylogenetic analysis inferred from an amino acid alignment of 64 plastid genes from streptophytes and green algae. The dataset was partitioned by gene according to the best-fit model assigned by PartitionFinder. Coloured vertical bars to the right of the phylogeny are labelled: S, Streptophytes; P, prasinophytes; CC, core Chlorophyta. Branch lengths are proportional to the number of substitution per site.

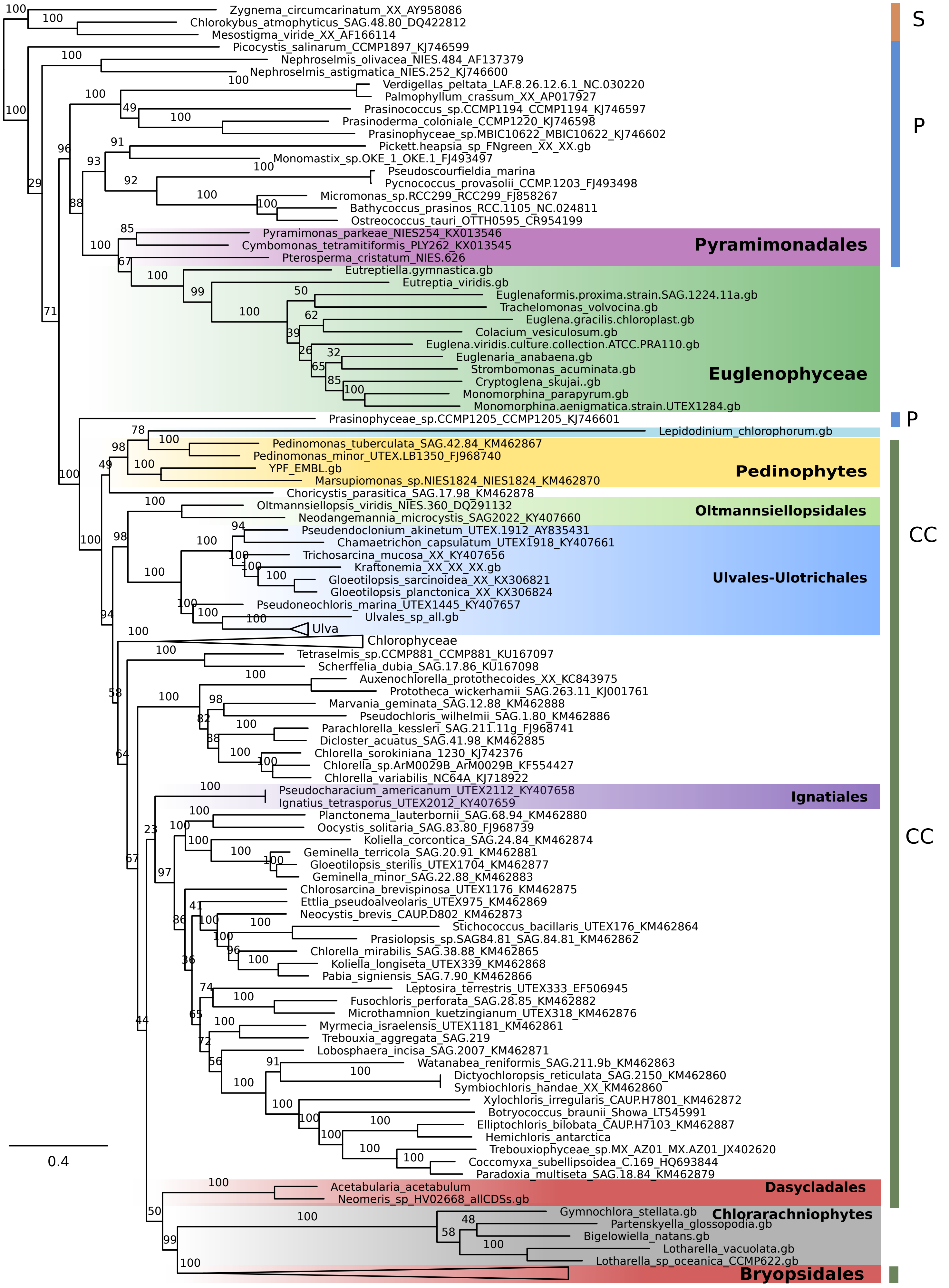

Figure S11. RAxML phylogenetic analysis (GTR model, partitioned codon position) inferred from a nucleotide alignment of 69 plastid genes (Dataset B; see methods) from streptophytes, green algae, photosynthetic euglenophytes, the "green" dinoflagellate *Lepidodinium chlorophorum*, and chlorarachniophytes. Coloured vertical bars to the right of the phylogeny are labelled: S, Streptophytes; P, prasinophytes; CC, core Chlorophyta. Branch lengths are proportional to the number of substitution per site.

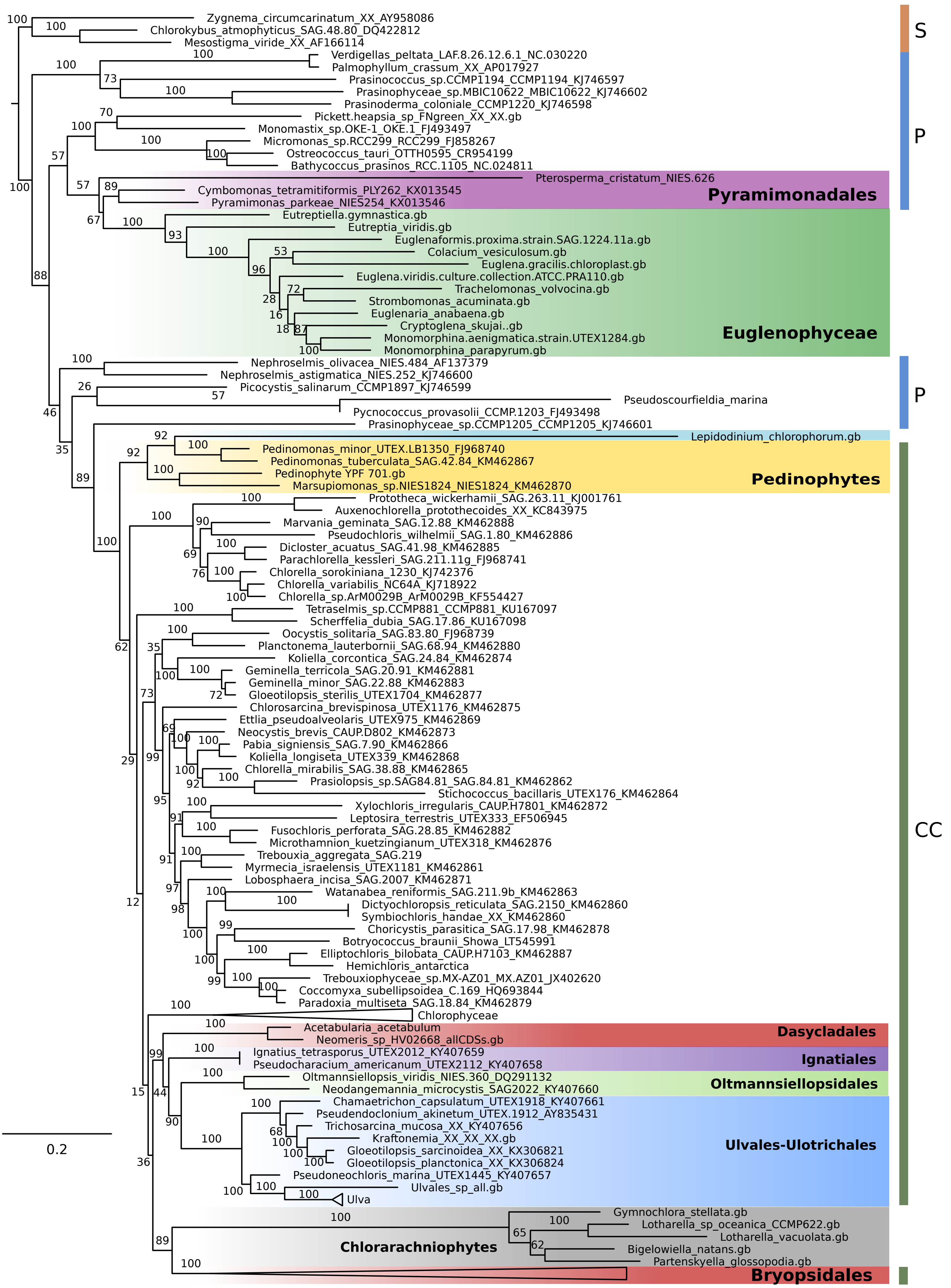

Figure S12. RAxML phylogenetic analysis (LG model) inferred from an amino acid alignment of 69 plastid genes (Dataset B; see methods) from streptophytes, green algae, photosynthetic euglenophytes, the "green" dinoflagellate *Lepidodinium chlorophorum*, and chlorarachniophytes. Coloured vertical bars to the right of the phylogeny are labelled: S, Streptophytes; P, prasinophytes; CC, core Chlorophyta. Branch lengths are proportional to the number of substitution per site.

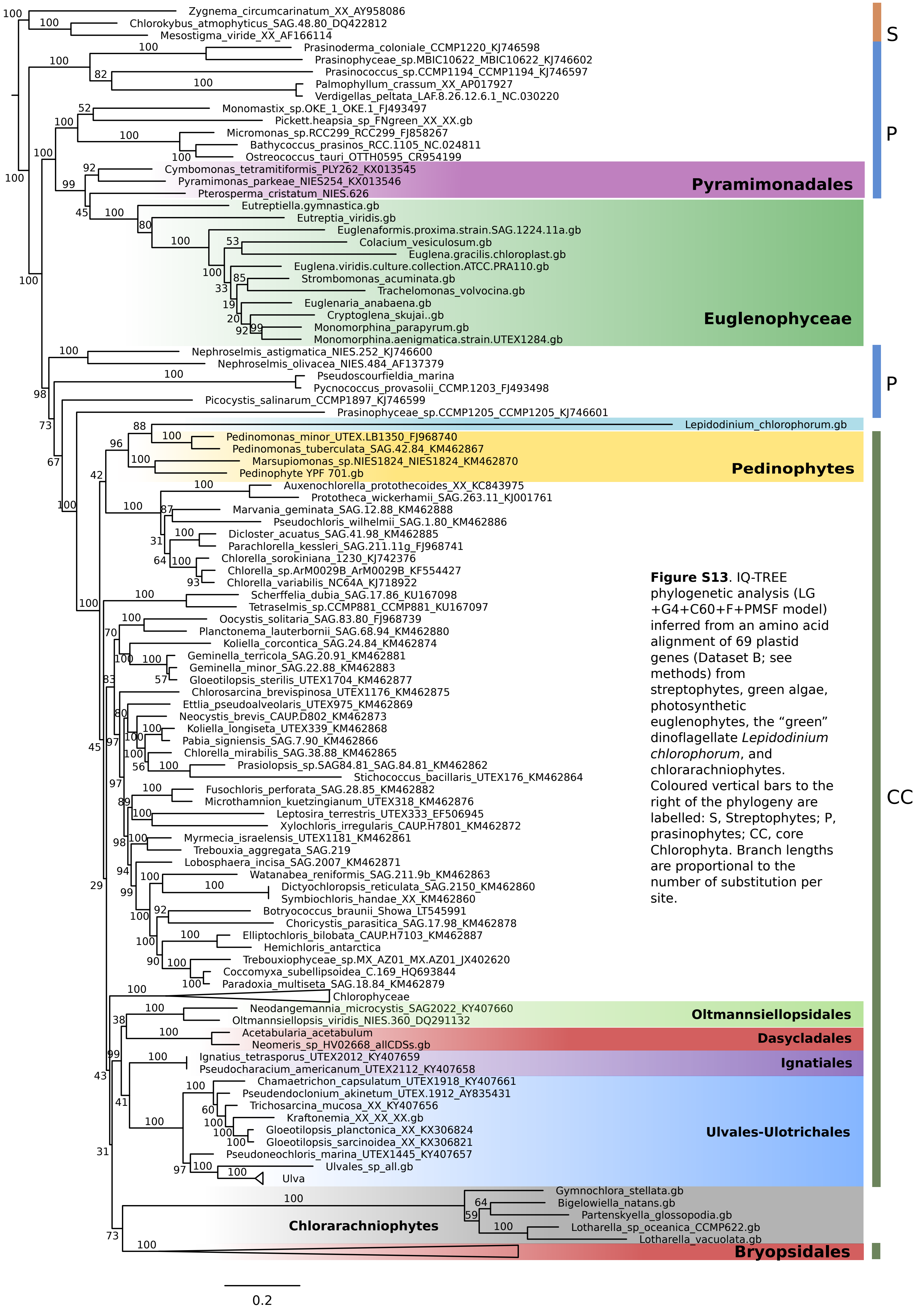

Figure S13. IQ-TREE phylogenetic analysis (LG +G4+C60+F+PMSF model) inferred from an amino acid alignment of 69 plastid genes (Dataset B; see methods) from streptophytes, green algae, photosynthetic euglenophytes, the “green” dinoflagellate *Lepidodinium chlorophorum*, and chlorarachniophytes. Coloured vertical bars to the right of the phylogeny are labelled: S, Streptophytes; P, prasinophytes; CC, core Chlorophyta. Branch lengths are proportional to the number of substitution per site.

Supplementary Information File S1.

Materials and Methods:

Relaxed molecular clock analyses.

Dataset assembly for molecular clock analysis

In order to perform molecular clock analyses we analysed single genes alignments from our 64-gene dataset using the 'gene shopping' method implemented in the software package SortaDate (Smith et al., 2017). Briefly, single-gene trees and alignments are compared and assessed for minimal conflict (i.e. they largely match a given species tree), lower root-to-tip variance, and discernible amounts of molecular evolution. Such genes behave in a more 'clock-like' manner, arguably reducing complications in divergence time-estimates caused by heterogeneity of rates among lineages and through time (Smith et al., 2017).

To run SortaDate we provided the species tree topology recovered from maximum likelihood analysis of our concatenated amino-acid dataset (Figure 1), amino-acid translations of each single-gene alignment, and manually rooted single gene trees. The setting '3,1,2' was used to rank genes: bipartition sorted first (3=bipartition), followed by root-to-tip variance (1=root-to-tip variance), and then tree length (2=tree-length). A concatenated dataset was compiled from the 11 top-ranked genes (*tufA*, *rps7*, *rps4*, *rpl14*, *rpl5*, *rpl2*, *psbC*, *psbB*, *petA*, *atpI*, *atpA*). In addition, we included the genes *atpB* and *rbcL*, due to availability of data from the taxa *Stauridium tetras*, *Pediastrum duplex* and *Hydrodictyon reticulatum*; these species belong to the Chlorophyceaen family Hydrodictyaceae, for which we have potential fossil records. Inclusion of the latter taxa therefore allowed an additional fossil calibration point (see below). In total, the concatenated dataset was 4083 amino-acids.

Node calibrations

To estimate the age of endosymbiosis events, we calibrated the phylogenetic tree in geological time using relaxed molecular clock methods. Node calibrations were derived from fossil information as well as node age estimates from previous studies. To our knowledge, no fossils are known for any of the lineages containing secondary green plastids, hence our node calibrations rely on fossil information for green algae (Table S1).

Table S1. Node calibrations used for relaxed molecular clock analysis

node	node	calibration	fossil or transfer	period	prior	reference
UA	Dasycladales-Bryopsidales split	UA_1	transfer from other study	n/a	U[631-511]	Verbruggen et al. 2009
UB	Ostreobium stem node	UB_1	transfer from other study	n/a	U[533-425]	Verbruggen et al. 2009
UC	BCD lineage stem node	UC_1	*Proterocladus*	Neoproterozoic	U[716-∞[	Butterfield et al. 1994
C	Hydrodictyaceae stem node	C_1	*Plaesiodictyon*	Triassic (Anisian)	U[237-∞[	Vigran et al. 1998
		C_2	*Hydrodictyolites*	Carboniferous	U[299-∞[	Elovski 1930
		C_3	*Deflandrastrum*	Silurian	U[416-∞[	Tappan 1980
		C_0	absence of calibration	n/a	n/a	n/a
T	Botryococcus stem node	T_1	*Botryococcus*	Carboniferous	U[299-∞[	Colbath & Grenfell 1995
		T_2	*Gloeocapsamorpha*	Ordovician	U[444-∞[	Traverse 1955
P	Pyramimonadales stem node	P_1	*Tasmanites*	Ediacaran	U[542-∞[	Arouri et al. 2000
RT	Streptophyta-Chlorophyta split	RT_1	transfer from other study	n/a	U[1279-1159]	Herron et al. 2009
		RT_2	transfer from other study	n/a	U[1015-863]	Parfrey et al. 2011

		RT_3	RT1 / RT2 hybrid	n/a	U[1279-863]	n/a
		RT_0	absence of calibration	n/a	n/a	n/a

Within the class Ulvophyceae, several reliable calibrations are available. A previous study used a range of fossils within the calcified siphonous green algae (Verbruggen et al., 2009), leading to well-defined ages for the split between the Dasycladales and Bryopsidales (calibration UA_1) and for the split between *Ostreobium* and the remaining Bryopsidales (UB_1). For these nodes, we defined a uniform prior range corresponding to the 95% HPD of node ages inferred by Verbruggen et al. (2009). The Neoproterozoic fossil *Proterocladus* (Butterfield et al. 1994) provides a third calibration point within the Ulvophyceae. *Proterocladus* is assigned to the siphonocladous green algae (order Cladophorales), which is not included in our analysis because no chloroplast genome data are available for them. This order was shown to be sister to the siphonous orders Dasycladales and Bryopsidales (Cocquyt et al. 2010), so we opted to calibrate the stem node of this lineage with *Proterocladus*, using a uniform prior with a lower bound of 700 Ma and no upper bound.

Within the class Chlorophyceae, some fossils are attributed to the family Hydrodictyaceae. We performed analyses with three different potential fossil Hydrodictyaceae genera to constrain the stem node of the family: the Triassic *Plaesiodictyon* (C_1), *Hydrodictyolites* (Carboniferous, C_2) and *Deflandrastrum* (Silurian, C_3). To assess the impact of these calibrations on node age estimates, we also ran an analysis where the node in question was unconstrained (C_0).

The trebouxiophycean genus *Botryococcus* is known with reasonable confidence from the Carboniferous (Colbath & Grenfell 1995), and we constrained its stem node with this age (T_1). It has been argued that the Ordovician *Gloeocapsamorpha* is also related to *Botryococcus* but this has been contradicted (Colbath & Grenfell 1995). We decided to experiment with this possibility by constraining the node to a minimum of 416 Ma (T_2).

Within the prasinophytes, the genus *Tasmanites* can be linked to the Pyramimonadales, and we used its Ediacaran age to constrain the stem node of the order (P_1).

Information is also available about the root node in our tree, which corresponds to the split between Streptophyta and Chlorophyta. This node was present in several previous relaxed molecular clock studies and we transferred the ages obtained there to our analysis. Herron et al. (2009) estimated the divergence at 1219 Ma (95% HPD: 1279-1159; calibration RT_1) while Parfrey et al. (2011) obtained a younger age of 939 Ma (95% HPD: 1015-863; calibration RT_2). We experimented with using either option, as well as a more agnostic hybrid between them, spanning from the oldest boundary from the older calibration to the youngest boundary from the younger calibration (1279-863; calibration RT_3). Finally, we also tried an analysis in which the root node was left unconstrained (RT_0).

Node age inference

All molecular clock analyses were run in PhyloBayes 4.1b (Lartillot et al. 2009), using the 13-gene amino-acid dataset. Two different models were tested: the lognormal autocorrelated clock (-ln option) and the uncorrelated gamma multipliers clock (-ugam option). Both models were run with either LG+Γ_4 or CATGTR+Γ_4. As shown in Table S10 below, divergence time estimates for nodes relevant to secondary green plastids (see Figure 2) were largely congruent between the models, with the exception of the chlorarachniophyte crown node. In the latter case the autocorrelated lognormal models estimated a much more recent date than the uncorrelated ugam models. However, as observed in this study (Figure 1) and others (Kamikawa et al., 2015; Suzuki et al., 2016; Tanifuji et al., 2014), branches leading to *Lepidodinium* and the chlorarachniophyte clade are long in comparison to their green algal relatives, suggesting that substitution rates have accelerated following secondary endosymbiosis. Therefore, to better account for the changes in molecular evolutionary rates that

appear to happen during/following secondary endosymbiosis, we opted to use the uncorrelated model (ugam with LG+Γ_4) rather than an autocorrelated model for our final molecular clock analysis. The tree topology was fixed (as required by PhyloBayes) using the phylogeny recovered from maximum likelihood analysis of amino-acid data (Dataset A; see main text for further discussion). We ran the MCMC analyses for 5,000 cycles, assessed convergence of likelihoods and parameter estimates in Tracer (Rambaut & Drummond 2009), and summarized the chains discarding the first 1,000 cycles as burn-in.

Table S10. Diverence date estimates for selected nodes using different molecular clock models

Model	Root	S/C	CC	Pyr/Eug	Eug crown	Lep/Ped	Bry/Chlora	Chlora crown
ugam+LG+Γ4	899	893	812	652	539	553	578	318
ugam+CAT+GTR+Γ4	890	885	813	635	509	564	597	282
ln+LG+Γ4	924	918	824	687	512	599	545	143
ln+CAT+GTR+Γ4	899	893	822	672	461	557	567	87

S/C = divergence of the Streptophyta and Chlorophyta; CC = divergence of the prasinophytes and core Chlorophyta; Pyr/Eug = divergence of the Pyramimonadales and the Eugleophytes; Eug crown = euglenophyte crown node; Lep/Ped = divergence of Lepidodinium and pedinophytes; Bry/Chlora = divergence of the Brypsidales and chlorarachniophytes; Chlora crown = chlorarachniophyte crown node.

Results from experimentation with different calibrations

As mentioned above, we experimented with various calibrations on particular nodes, and various combinations of those calibrations. This showed that especially the two root calibrations transferred from previous studies (RT_1 vs. RT_2) had a profound effect on inferred node ages, with ages along the backbone of the tree being more than 100 Ma older for RT_1 than RT_2. When no root calibration was used (RT_0), the remaining constraints resulted in an estimated root node age of ~894 Ma, which is more in line with the Parfrey et al. 2011 study than with the Herron et al. 2009 study. Similarly, the calibration that spanned from the oldest boundary of the older calibration to the youngest boundary of the younger calibration (RT_3), we obtained an average root age of ~900 Ma.

The different calibrations for *Botryococcus* (T_1 vs. T_2) had some influence within the Trebouxiophyceae clade but did not affect other branches much. Because the branch leading to the *Botryococcus* with the T_2 calibration was much longer than it was without any calibration on the node, and because the evidence for a eukaryotic nature of *Gloeocapsamorpha* has been contradicted, we considered this to be an unlikely calibration and opted for T_1 instead.

The calibration of the stem node of Hydrodictyaceae (C) had influence primarily within the Chlorophyceae clade but also changed ages at deeper nodes in the tree by about 10 Ma. Not surprisingly, the older the calibration, the more node ages were pushed back. What was most interesting about our experiments with node C is the finding that without any calibration (C_0), the stem node of Hydrodictyaceae was estimated to be merely 137 Ma, considerably younger than the fossils assigned to the family.

The conclusions of our experiments with different combinations of node calibrations are that (1) root node calibrations agree except for RT_1, (2) *Botryococcus* calibration T_1 appears to be the most reasonable, (3) none of the calibrations on the Hydrodictyaceae appear reliable, (4) the remaining calibrations do not appear to contradict one another.

Presenting the final chronogram

Following the conclusions from the experimentation, we opted to present the results from the analysis with calibrations C_0, T_1 and RT_3, as well as the calibrations for the remaining 4 nodes (P_1, UA_1, UB_1, UC_1). In other words, we opted not to use any presumed Hydrodictyaceae fossils, to use the Carboniferous date to constrain the Botryococcus lineage, and to apply a root age prior spanning the results from both previous studies.

Our final chronogram recovers an estimated age of ~900 Ma for the root node, that is, the split between the Streptophyta and Chlorophyta (Figure 2, S/C split), with a 95% confidence interval (95 % CI) of ~865 – 958 Ma. This younger boundary is in line with that recovered by Parfrey et al. (2011) (863 Ma), whereas the older boundary is younger than that recovered by Herron et al. (2009) (1159 Ma).

Radiation of the Chlorophyta, corresponding to the divergence of prasinophyte lineages, is dated at ~894 Ma (95% CI ~859 – 953]. Radiation of the prasinophyte order Pyramimonadales, the lineage most closely related to the Euglenophyceae plastid, is dated at ~710 Ma (95% CI ~621 – 790). The core Chlorophyta diverge from the prasinophyte lineages at ~812 Ma (95% CI ~781 – 859 Ma), with radiation of the core Chlorophyta lineages at ~770 Ma (~746 – 806). Radiation of the pedinophytes, the lineage most closely related to the *Lepidodinium* plastid, is dated at ~672 Ma (95% CI ~560 – 747).

Divergence dates of secondary green plastid lineages from their green algal sister lineages were as follows. The euglenophyte plastid lineage split from the prasinophyte order Pyramimonadales ~652 Ma, with Euglenophyceae radiation beginning ~539 Ma. The 95% confidence intervals for these nodes overlap (95% CI ~563 – 728 and ~453 – 631, respectively). The *Lepidodinium chlorophorum* plastid lineage diverged from the other pedinophytes ~553 Ma, with a 95% CI of ~416 – 661. Finally, the chlorarachniophyte plastid lineage diverged from the siphonous green algal order Bryopsidales ~578 Ma (95% CI ~546 – 603), with chlorarachniophyte radiation dated at ~318 Ma (95% CI ~250 – 388). As discussed in the main text, it is not possible to determine at which point during this proposed timeframe secondary endosymbiosis took place.

Site-wise likelihood analyses.

Following the methodology of Shen et al. (2017), we measured the phylogenetic signal of each site in our nucleotide alignment to quantify site-wise support for the tree topology with the Dasycladales+chlorarachniophyte+Bryopsidales clade (hereafter referred to as topology T1, as shown in Figure S1) vs support for the topology containing the Dasycladales+ Oltmannsiellopsidales+Ulvales-Ulotrichales clade (topology T2, as shown in Fig. 1). See Table S2 for results.

To compare the support of each codon position in our alignment for topology T1 or T2, we examined sites from each position separately. The majority of first and second-position sites supported topology T1 (69% and 77%, respectively, Table S3). However, the combined signal from these sites was somewhat weaker than the combined signal of first and second position-sites favouring topology T2 (31% and 23% of sites, respectively). In terms of third-position sites, a slight majority supported topology T1 (58%), and the combined signal of these was much greater than those third-position sites supporting topology T2 (Table S3).

Given that most substitutions at third codon positions are synonymous, the strong signal supporting topology T1 at these sites is largely obscured in the translated amino-acid dataset, likely accounting for the differing topologies recovered using nucleotide vs amino-acid data. Consistent with this observation, ML analysis of a nucleotide dataset with third codon-positions removed recovered an overall topology consistent with T2; chlorarachniophytes branch with the Bryopsidales with full support, and Dasycladales branch with the Oltmannsiellopsidales+Ulvales-Ulotrichales lineages (80%

BS) (Figure S5). Both these clades branch together with moderately strong support (BS 84%), and this relationship is also weakly recovered in our ML analysis of amino-acid data (BS 40%, Figure 1). Moreover, progressive removal of fast-evolving sites from our full nucleotide alignment, followed by ML analyses, showed that support for a Dasycladales+chlorarachniophyte+Bryopsidales clade was high (>90% BS) for the 95, 90, 85, and 80% datasets, before dropping to 69% BS for the 75% dataset. Conversely, Dasycladales branched as sister to the Oltmannsiellopsidales+Ulvales-Ulotrichales clade with strong to very strong support in the 70, 65, 60, and 55% datasets (Figure S8, Table S8). As substitution rates are likely to be highest at third codon positions, these sites are likely among the first to be removed in our analyses; the differing branching position of the Dasycladales in the 70 – 55% datasets is therefore consistent with the analysed described above.

Given the overall strong support of third-position sites for topology T1, we were interested to see if this pattern was reflected in putative shared codon usage biases in the Dasycladales and Bryopsidales, to the exclusion of the Oltmannsiellopsidales+Ulvales-Ulotrichales lineages branching with Dasycladales in topology T2. To assess only alignment sites with data from all species, all alignment columns with gaps were removed from our nucleotide dataset, and codon usage was calculated from the remaining 14,079 positions. No clear patterns could be discerned (see Table S6), perhaps suggesting that relatively subtle biases in codon usage can lead to significantly different site-wise phylogenetic biases in our dataset.

Finally, we also calculated site-wise support for topology T1 vs T2 based on our amino-acid alignment, and used these data to assess per-gene support (Table S4 – S5). As expected, the majority of genes supported topology T2 at the amino-acid level (Table S5). In comparison, ~50% of genes supported topology T1 at the nucleotide level; consistent with the analyses above, the signal from genes supporting T1 was much overall much greater than the genes supporting T2 (Table S5).

References:

Arouri, K. R., Greenwood, P. F. and Walter, M. R. 2000. Biological affinities of Neoproterozoic acritarchs from Australia: Microscopic and chemical characterisation, Organic Geochemistry, 31(1), pp. 75–89. doi: 10.1016/S0146-6380(99)00145-X.
Butterfield, N. J., Knoll, A. H. & Swett, K. 1994. Paleobiology of the Neoproterozoic Svanbergfjellet Formation, Spitsbergen. *Fossils and Strata* **34**: 1–84.
Colbath, G. K. & Grenfell, H. R. 1995. Review of biological affinities of Paleozoic acid-resistant, organic-walled eukaryotic algal microfossils (including acritarchs). *Review of Palaeobotany and Palynology* **86**: 287–314.
Elovski, V.A., 1930. Microscopical structure of the coal seam Moschny, Chernogorski Mines, Minusinsk Basin. *Tr. Gl. Geol.-razved. Upr. Vys. Sov. Narod. Choz. SSSR*, **4**: 7–39.
Herron, M. D., Hackett, J. D., Aylward, F. O. & Michod, R. E. 2009. Triassic origin and early radiation of multicellular volvocine algae. *Proceedings of the National Academy of Sciences of the United States of America* **106**:3254–58.
Kamikawa R, Tanifuji G, Kawachi M, Miyashita H, Hashimoto T, Inagaki Y. 2015. Plastid genome-based phylogeny pinpointed the origin of the green-colored plastid in the dinoflagellate *Lepidodinium chlorophorum*. Genome Biology and Evolution **7**: 1133–1140.
Lartillot, N, Lepage, T. & Blanquart, S. 2009. PhyloBayes 3: a Bayesian software package for phylogenetic reconstruction and molecular dating. *Bioinformatics* **25**:2286–88.
Parfrey LW, Lahr DJG, Knoll AH, Katz L a. 2011. Estimating the timing of early eukaryotic diversification with multigene molecular clocks. *Proceedings of the National Academy of Sciences of the United States of America* **108**: 13624–9.
Pond, S. L. K., Frost, S. D. W. and Muse, S. V. 2005. HyPhy: hypothesis testing using phylogenies, *Bioinformatics (Oxford, England)*, **21**(5), pp. 676–9. doi: 10.1093/bioinformatics/bti079.
Rambaut, A. & Drummond, A. J. 2009. Tracer. http://beast.bio.ed.ac.uk/tracer.
Shen X, Hittinger CT, Rokas A. 2017. Contentious relationships in phylogenomic Studies Can Be Driven By a Handful of Genes. Nature Publishing Group **1**: 1–10.

Smith SA, Brown JW, Walker JF. 2017. So many genes, so little time : comments on divergence-time estimation in the genomic era. bioRxiv preprint doi: https://doi.org/10.1101/114975
Suzuki S, Hirakawa Y, Kofuji R, Sugita M. 2016. Plastid genome sequences of *Gymnochlora stellata*, *Lotharella vacuolata*, and *Partenskyella glossopodia* reveal remarkable structural conservation among chlorarachniophyte species. *Journal of Plant Research* **129**: 581–590.
Tanifuji G, Onodera NT, Brown MW, Curtis B a, Roger AJ, Ka-Shu Wong G, Melkonian M, Archibald JM. 2014. Nucleomorph and plastid genome sequences of the chlorarachniophyte *Lotharella oceanica*: convergent reductive evolution and frequent recombination in nucleomorph-bearing algae. *BMC genomics* **15**: 374.
Tappan, H. 1980. The paleobiology of plant protists. Freeman, San Francisco, 1028 pp.
Traverse, A. 1955. Occurrence of the oil-forming alga Botryococcus in lignites and other Tertiary sediments. Micropaleontology **1**: 343-350.
Verbruggen H, Ashworth M, LoDuca ST, Vlaeminck C, Cocquyt E, Sauvage T, Zechman FW, Littler DS, Littler MM, Leliaert F, et al**. 2009**. A multi-locus time-calibrated phylogeny of the siphonous green algae. *Molecular Phylogenetics and Evolution* **50**: 642–653.
Vigran, J.O., Mangerud, G., Mork, A., Bugge, T. & Weitschat, W. 1998. Biostratigraphy and sequence stratigraphy of the Lower and Middle Triassic deposits from the Svalis Dome, Central Barents Sea, Norway. *Palynology* **22**: 89-141.
